# Phenotypic Consequences of *SLC25A40-ABCB1* Fusions beyond Drug Resistance in High-Grade Serous Ovarian Cancer

**DOI:** 10.3390/cancers13225644

**Published:** 2021-11-11

**Authors:** Kathleen I. Pishas, Karla J. Cowley, Ahwan Pandey, Therese Hoang, Jessica A. Beach, Jennii Luu, Robert Vary, Lorey K. Smith, Carolyn E. Shembrey, Nineveh Rashoo, Madelynne O. White, Kaylene J. Simpson, Andrea Bild, Jason I. Griffiths, Dane Cheasley, Ian Campbell, David D. L. Bowtell, Elizabeth L. Christie

**Affiliations:** 1 Peter MacCallum Cancer Centre, Melbourne, VIC 3000, Australia; kathleen.pishas@petermac.org (K.I.P.); ahwan.pandey@petermac.org (A.P.); therese.hoang@petermac.org (T.H.); jessia.beach@education.vic.gov.au (J.A.B.); lorey.smith@petermac.org (L.K.S.); cshembrey@student.unimelb.edu.au (C.E.S.); nineveh.rashoo@petermac.org (N.R.); madelynne.white@petermac.org (M.O.W.); dane.cheasley@petermac.org (D.C.); ian.campbell@petermac.org (I.C.); david.bowtell@petermac.org (D.D.L.B.); 2 The Sir Peter MacCallum Department of Oncology, The University of Melbourne, Melbourne, VIC 3010, Australia; kaylene.simpson@petermac.org; 3 Victorian Centre for Functional Genomics, Peter MacCallum Cancer Centre, Melbourne, VIC 3000, Australia; karla.cowley@petermac.org (K.J.C.); Jennii.Luu@petermac.org (J.L.); robert.vary@petermac.org (R.V.); 4 Department of Clinical Pathology, Faculty of Medicine, Dentistry, and Health Science, The University of Melbourne, Melbourne, VIC 3000, Australia; 5 Department of Medical Oncology and Therapeutics Research, City of Hope National Medical Center, Duarte, CA 91010, USA; abild@coh.org (A.B.); jasonigriff@gmail.com (J.I.G.)

**Keywords:** *ABCB1*, high-grade serous ovarian cancer, drug resistance, high-throughput drug screening

## Abstract

**Simple Summary:**

Among the plethora of malignancies affecting the female reproductive tract, those concerning the ovary are the most frequently fatal. In particular, chemotherapy-resistant High-Grade Serous Ovarian Cancer (HGSOC) remains a clinically intractable disease with a high rate of mortality. We previously identified *SLC25A40-ABCB1* transcriptional fusions as the driving force behind drug resistance in HGSOC. As success in the clinical arena will only be achieved by enhancing our fundamental understanding of the drivers that mediate cellular drug resistance, this report sought to elucidate the phenotypic, metabolomic and transcriptional consequences of *SLC25A40-ABCB1* fusions beyond drug resistance. High-throughput FDA drug screening was also undertaken to identify new therapeutic avenues against drug-resistant cellular populations.

**Abstract:**

Despite high response rates to initial chemotherapy, the majority of women diagnosed with High-Grade Serous Ovarian Cancer (HGSOC) ultimately develop drug resistance within 1–2 years of treatment. We previously identified the most common mechanism of acquired resistance in HGSOC to date, transcriptional fusions involving the ATP-binding cassette (ABC) transporter *ABCB1*, which has well established roles in multidrug resistance. However, the underlying biology of fusion-positive cells, as well as how clonal interactions between fusion-negative and positive populations influences proliferative fitness and therapeutic response remains unknown. Using a panel of fusion-negative and positive HGSOC single-cell clones, we demonstrate that in addition to mediating drug resistance, *ABCB1* fusion-positive cells display impaired proliferative capacity, elevated oxidative metabolism, altered actin cellular morphology and an extracellular matrix/inflammatory enriched transcriptional profile. The co-culture of fusion-negative and positive populations had no effect on cellular proliferation but markedly altered drug sensitivity to doxorubicin, paclitaxel and cisplatin. Finally, high-throughput screening of 2907 FDA-approved compounds revealed 36 agents that induce equal cytotoxicity in both pure and mixed *ABCB1* fusion populations. Collectively, our findings have unraveled the underlying biology of *ABCB1* fusion-positive cells beyond drug resistance and identified novel therapeutic agents that may significantly improve the prognosis of relapsed HGSOC patients.

## 1. Introduction

*“The law is not the survival of the “better” or the “stronger”. It is the survival of those which are constitutionally fittest to thrive under the conditions in which they are placed.”*

Herbert Spencer

Despite our rapidly evolving knowledge concerning cancer cell heterogeneity, we are far from deciphering the complex dynamics that operate between cellular subpopulations within tumors. Indeed, most of the conventional cytotoxic drug-based strategies which still dominate anticancer treatment do not account for target cell heterogeneity and have reached their limit in terms of efficacy. As growing evidence suggests that cancer cells behave as communities, increasing attention is now being directed towards understanding how the intense selection pressures imposed by cytotoxic therapies influences clonal evolutionary dynamics and thus therapeutic response. With each cubic centimeter of tumor containing up to one billion cancer cells [1], new insights into how clonal interactions and evolutionary dynamics between drug-sensitive and drug-resistant cellular populations influences tumorigenesis and disease progression are urgently required to substantially change therapeutic treatment practices and shift overall survival. This is particularly pertinent for women diagnosed with HGSOC.

To date, ovarian cancer remains the deadliest form of gynecological malignancy, with >140,000 women globally succumbing to disease each year [2,3]. Of these, the HGSOC histosubtype predominates clinical settings and is responsible for a disproportionate share of fatalities from all forms of ovarian cancer (70–80%) [4]. This high mortality is largely due to a lack of effective screening and early detection methods leading to advanced disease at presentation and resistance to available treatments. These genetically highly unstable tumors are characterized by a high degree of genomic copy number change rather than recurrent point mutation, with only *TP53* frequently mutated (96.7%) [5,6,7]. As such, the dearth of targetable mutations, with the exception of PARP (poly ADP ribose polymerase) inhibitors for BRCA1/2 mutant cohorts [8], has not fueled profound improvements in overall survival outcomes over the past few decades (overall survival of 40.7 months) [9].

The invariable emergence of drug resistance following initial response to chemotherapy and limited molecularly targeted approaches continue to be the principal limiting factor to achieving cures in patients with cancer. Current HGSOC chemotherapy regimens rely on platinum and taxane-based agents. However, despite initial sensitivity to first-line chemotherapy, 25% of patients will become platinum-refractory in the primary setting, with 20% becoming platinum-resistant with a recurrence within six months of the conclusion of chemotherapy [10,11]. Our recent extensive genomic characterization of post treatment HGSOC patient samples identified the most common mechanism of acquired resistance in HGSOC to date, transcriptional fusions involving the ATP-binding cassette (ABC) efflux transporter *ABCB1* (also known as MDR1), which has well established roles in multidrug resistance (MDR) [12], and the solute carrier *SLC25A40* [7,13]. Indeed, *ABCB1* chromosomal rearrangements driving MDR have also been detected in ALL (acute lymphocytic leukemia) [14] and breast cancer [13]. These resulting fusions place *ABCB1* under the control of a strong promoter whilst leaving its open reading frame intact [7,13].

ABC transporters differ from classical selective transporters by way of their promiscuity for structurally and chemically diverse substrates (>200) and contribute to tumor biology independently of their ability to efflux cytotoxic drugs [15]. Indeed, this family of 48 transporters is also involved in lipid export/homeostasis and mediates the release of bioactive lipids (phospholipids and sphingolipids) that activate signaling cascades involved in cellular proliferation, migration and tumorigenesis [16,17]. *ABCB1*, which encodes the drug efflux transporter P-glycoprotein (P-gp), is the most extensively studied member of the ABC transporter superfamily. *ABCB1* couples the hydrolysis of ATP (adenosine triphosphate) to export numerous xenobiotics and toxic metabolites (cationic hydrophobic compounds (300–4000 Da) [18] in a unidirectional path across the phospholipid bilayer of cellular membranes against a chemical gradient [19], including agents that are central to most chemotherapeutic regimens, including anthracyclines (doxorubicin), tyrosine kinase inhibitors (imatinib), taxanes (paclitaxel), epipodophyllotoxins (etoposide) and vinca alkaloids (vincristine) [12,20]. Indeed, *ABCB1* fusion events were only detected in HGSOC patients who had been exposed to known *ABCB1* substrate chemotherapies (doxorubicin and paclitaxel), with the probability of a fusion event being closely correlated to the number of lines of substrate chemotherapy administered [13]. Interestingly, our genomic analysis revealed that not all cells within the tumor population harbored *ABCB1* fusions, raising the question of how fusion-negative cells have survived under chemotherapy-induced selective pressure. Besides the high energy expenditure required to actively export drugs or xenobiotics, the phenotypic consequences of *ABCB1* overexpression beyond drug extrusion and how clonal cooperation/dynamics between *ABCB1* fusion-positive and negative cells influences cellular behavior, tumor fitness and therapeutic response remains unknown.

Although P-gp was first described in 1976 [21], the potential benefit of inhibiting *ABCB1* transporter activity and thus reversing therapy resistance has not come to clinical fruition despite the wealth of knowledge concerning the biochemistry and substrate specificity of ABC transporters. The shortcomings of several third generation P-gp inhibitors in the clinic have primarily been attributed to their distribution to non-target organs, as P-gp is constitutively expressed on epithelial cells of the kidney, liver, pancreas and intestine [22], leading to intolerable side effects [23]. As the emergence of therapy resistance is generally viewed as an evolutionary process in which cancer cells adapt to selection pressures mediated by cytotoxic drugs, this study has specifically focused on elucidating the underlying biology of drug-resistant *ABCB1* fusion-positive cells and how this distinctive phenotype, in combination with evolutionary dynamics, can be clinically exploited.

Using a panel of single-cell *SLC25A40-ABCB1* fusion-negative and positive clones, our study sought to elucidate the complex underlying biology of fusion-positive cells beyond drug resistance as well as identify therapeutic options that can be utilized to combat this nefarious population of cells.

## 2. Materials and Methods

### 2.1. Cell Line Culture

Patient-derived HGSOC cell lines AOCS18.5 and AOCS21.2 were established from AOCS (Australian Ovarian Cancer Study) patient ascites as previously described [13]. CAOV3, JHOS2 and Caco-2 cell lines were purchased from American Type Culture Collection (ATCC). Cell lines were grown in RPMI 1640 (GIBCO, Carlsbad, CA, USA), media supplemented with 10% HyClone Fetal Bovine Serum (GE Healthcare, Chicago, IL, USA) and 1% Penicillin-Streptomycin-Glutamine (GIBCO). Cell lines were tested yearly for Mycoplasma infection (Genotyping Core, Peter MacCallum Cancer Centre, Melbourne, Australia).

Whole genome sequencing (WGS) of the parental AOCS18.5 line revealed a *SLC25A40-ABCB1* variant allele frequency (VAF) of 0.2, suggesting that approximately 40% of the AOCS18.5 cellular population harbored a heterozygous ~250 kb deletion, as previously described [7,13], leading to the *SLC25A40-ABCB1* transcriptional fusion. To generate AOCS18.5 single-cell fusion-negative and positive clones, the parental line was seeded at low density (50–100 cells) onto 10 cm dishes and grown for 5 days with clones isolated using 3.22 mm cloning discs (Sigma-Aldrich, St. Louis, MO, USA). Fusion status was confirmed through fusion specific qRT-PCR [13] and breakpoint specific PCR (DNA detection primers: Forward 5′GTG GTC CCC GCC TGT AAC′3, Reverse 5′GTG GTC CAT CTG GGG TAA ATG′3).

AOCS18.5 Clone D (*ABCB1* fusion-negative) and Clone 9 (*ABCB1* fusion-positive) cells were transduced with plasmids of Lentiviral Gene Ontology (LeGO) vectors encoding either Venus Yellow (Addgene plasmid 27340) or mCherry (Addgene plasmid 27339) as previously described [24]. The top 50% of Venus Yellow and mCherry cells were collected through fluorescence-activated cell sorting (FACS) analysis. Vectors were kindly provided by Dr. Carolyn Shembrey (Peter MacCallum Cancer Centre, Melbourne, Australia) and Prof Frederic Hollande (The University of Melbourne, Melbourne, Australia).

### 2.2. Immunodetection

Whole-cell lysates were prepared using RIPA lysis buffer containing protease inhibitor cocktail (Roche) followed by sonication. Protein concentrations were determined using the Pierce BCA Protein Assay Kit (Thermo Fisher Scientific, Waltham, MA, USA). In total 40 µg of protein was loaded onto Mini-PROTEAN TGX 4–20% gels (Bio-Rad, Hercules, CA, USA) and subjected to gel electrophoresis at 90 V for 10 min followed by 150 V for 90 min and was then transferred onto nitrocellulose membranes using an iBlot2 (Thermo Fisher Scientific, Waltham, MA, USA) according to the manufacturer’s instructions. Membranes were blocked in Odyssey Blocking Buffer (PBS; LI-COR Bioscience) for 1 h at room temperature followed by overnight 4 °C incubation with primary antibodies, GAPDH (Ab8245, 1:10,000) and MDR1 (Ab170904, 1:1000). Immunodetection was achieved after incubation with infrared (IR) dye-conjugated 800CW secondary antibodies (LiCor) with bands visualized using the Odyssey Imaging System. Densitometry analysis was performed using ImageJ software (V.153k), with ABCB1 protein normalized to GAPDH loading control and AOCS18.5 parental cells. 

### 2.3. IncuCyte Live Cell Imaging

Cells were seeded (1000–3000 cells/well) in 96 flat clear or black wall (fluorescence assays only) microtiter plates and left to adhere overnight (triplicate wells per condition). Phase contrast and/or green/red fluorescent images (10× magnification) were taken in the IncuCyte ZOOM or SX5 Imaging System (Sartorius, Göttingen, Germany) at 12 or 24 h intervals for a maximum of 264 h, with media changed every 96 h. Cell confluence (phase contrast) or green/red (800 ms) fluorescence area (µm^2^/Image) was evaluated using IncuCyte ZOOM 2016A software (Sartorius). For our purposes, a cell was defined as an entity with an area greater than 450 μm^2^. Data represents mean ± SEM from a minimum of 4 independent experiments.

### 2.4. MDR Efflux Assay

The multidrug resistance direct dye efflux assay was performed according to the manufacturer’s protocol (Merck Millipore, Burlington, MA, USA). Briefly, 2.5 × 10^5^ cells per cell line were collected and incubated with Rhodamine 123 loading buffer (1 h at 4 °C). Cells were centrifuged (200× *g*, 5 min), resuspended in cold efflux buffer and separated into 3 treatment groups: (i) 37 °C warmed efflux buffer containing DMSO, (ii) 37 °C warmed efflux buffer containing Vinblastine and (iii) ice-cold efflux buffer. Cells were incubated for 3 h at either 37 °C or 4 °C, followed by 2 washes in ice cold efflux buffer. Cells were then stained with FluoroGold (1:300, Sigma-Aldrich) and assessed for Rhodamine 123 efflux through Flow Cytometry Analysis (FACS) analysis (10,000 cells).

### 2.5. RNA Extraction and qRT-PCR

Total RNA was extracted using the RNeasy kit (Qiagen, Hilden, Germany) with on-column DNase digestion. cDNA was generated using 1000 ng of total RNA (Bioline SensiFast cDNA synthesis kit, (Meridian Bioscience, Cincinnati, OH, USA) with subsequent qRT-PCR performed using SYBR Green PCR mix (Applied Biosytems, Waltham, MA, USA). Reactions were processed on a LightCycler 480 (Roche, Basel, Switzerland) with subsequent gene expression quantified using the ΔΔCT method from triplicate reactions. *ABCB1* gene expression and *SLC25A40-ABCB1* fusion expression were normalized to the internal housekeeping genes *GAPDH* and *HPRT*. Primer sequences as previously described [13].

### 2.6. RNA Sequencing

For cell line analysis, 1 μg of total RNA was submitted for NextSeq 100 bp paired-end, polyA RNA sequencing (Molecular Genomics Core, Peter MacCallum Cancer Centre), 3 independent replicates per clone. Libraries were prepared using the NEBNext Ultra II Directional RNA Library Prep Kit for Illumina, generating 20 million paired reads per sample. Reads were mapped to the human reference GRCh37.92 using the STAR 2-pass method (v2.6.0b). Counts were generated on the ensemble release GRCh37.92 GTF annotation using HTSeq (v0.10.0). Counts were normalized to logged TMM values using edgeR (v3.28.1). TPM expression values were also generated. Differential gene expression analysis was performed using DESeq2 (v1.26.0) on the raw counts. The fGSEA R package (v1.15.1) was run on the genes ranked by DESeq2 log_2_ fold changes for the MSigDB Hallmark Pathways (v7.1), generating normalized enrichment scores (NES) for each pathway. Fusion status was confirmed using the Arriba fusion tool (v1.1.0).

Expression profiles from relapsed fusion-negative (*n* = 21) and fusion-positive (*n* = 6) HGSOC patient’s samples were previously conducted [7,13]. Clinical characteristics of the HGSOC patient cohort are detailed in Christie et al. (2019) and Patch et al. (2015) [7,13]. Women diagnosed with HGSOC were recruited at hospitals across Australia and were recruited through the AOCS. Ethics board approval was obtained at all institutions for patient recruitment, sample collection and research studies (HREC protocol 01/60 and 16/161). Written informed consent was obtained from all participants.

### 2.7. Extracellular Flux Analysis

Extracellular flux analyses were performed on a Seahorse XFe96 Analyzer (Agilent, Santa Clara, CA, USA). Assay medium was prepared using Seahorse XF Base Medium (containing 5.5 mM glucose, 2 mM glutamine and 1 mM sodium pyruvate, adjusted to pH 7.4 and kept at 37 °C; Agilent 102353-100).

The XF Cell Mito Stress Test was performed as previously described [25]. Cells were seeded on Seahorse XF 96 well cell culture plates (5000–14,000 cells per well) 48 h prior to analysis (quadruplicate wells per condition). Cell culture medium was removed and replaced with Seahorse XF medium, with cells equilibrated in a non-CO_2_ incubator for 1 h prior to the assay. The XF Cell Mito Stress Test protocol was performed as per manufacturer’s directions, using oligomycin (1 μM), FCCP (1 μM) and rotenone/antimycin A (1 μM). The assay was run with repeated cycles of 3 min mix and 3 min measurements following each drug injection with simultaneous measurement of oxygen consumption rate (OCR) and extracellular acidification rate (ECAR). At completion of the assay, cells were injected with Hoescht live-cell nuclear stain and imaged using a Cellomics Arrayscan automated microscope (10× magnification; 4× fields). OCR and ECAR values were subsequently normalized to cell number, and data were analyzed using the Mito Stress Test Report Generator (Agilent).

### 2.8. High-Throughput Drug Screening

AOCS18.5 clones (D, E, 9,18B and 50:50 Clone D:9 mix) were seeded into 384 Corning plates (Cat #3904) (1000–2000 cells per well) and left to adhere overnight. For initial primary screening (2907 compounds), cell lines were treated with a 3-point dose curve (5, 0.5, 0.05 µM). For the validation screen, cell lines were treated with a 6-point dose curve (10, 5, 2.25, 0.5, 0.225, 0.05 µM) (duplicate wells). The open access FDA drug library was sourced from Compounds Australia (Griffith University, Australia) and covered a diverse spectrum of drug classes, mode of actions and targets. Following 72 h of treatment, cells were fixed in 4% paraformaldehyde (10 min; ProSciTech, Townsville, Australia), washed with PBS and then stained with a 0.19% Triton X solution containing DAPI (4′,6-diamidino-2-phenylindole) (Nuclear) (Sigma-Aldrich, St. Louis, MO, USA)) (1:1000), CellMask Green (Plasma membrane) (Thermo Fisher Scientific, Waltham, MA, USA) (1:20,000) and Rhodamine/Phalloidin (F-actin filaments) (Biotium, Fremont, CA, USA) (1:500). All automated liquid handling was performed at the Peter MacCallum Cancer Centre VCFG (Victorian Centre for Functional Genomics) core. Automated drug dispensing was performed using the Sciclone ALH 3000 Workstation (PerkinElmer, Waltham, MA, USA) with end staining facilitated with the BioTek workstation (BioTek Instruments, Winooski, VT, USA).

### 2.9. Viability and Actin Morphology

For cell viability, the entire area of each well was imaged (9 fields) at 10× magnification, and for actin morphology, ~300 cells were imaged at 20× magnification using a Cell Insight CX7 High-Content Screening (HCS) Platform (Thermo Fisher Scientific, Waltham, MA, USA). The images were then analyzed for nuclear count and actin morphology using a CellProfiler v3.0 pipeline run on a high-performance computer cluster. To identify cell viability hits, the cell counts were first normalized for batch effects by fold change to the median of the negative control wells on a per plate basis. The normalized values were then robust Z-scored to the entire dataset in order to determine the relative strength of each compound.

To compare actin morphology between fusion-positive and negative clones, 148 measurements encompassing a wide range of features related to cell shape and actin intensity and texture were extracted from untreated cells. These raw values were first normalized and scaled to a reference distribution by robust Z-Scoring each feature to the median and median absolute deviation of the same feature in the fusion-negative cell population. The entire dataset was then passed through an iterative feature reduction process. Features with low variance across fusion-positive and fusion-negative clones, or inactive features, as well as highly correlated (Pearson’s correlation coefficient > 0.85) or redundant features, were removed. Upon completion of this process, a final set of 59 features was selected for further analysis.

### 2.10. Targeted Sequencing

Targeted sequencing was performed on AOCS18.5 clone DNA using a customized Agilent SureSelect XT Low Input capture panel (Design ID 3221041) and the SureSelect XT Low Input Target Enrichment System (Agilent). Libraries were sequenced on an Illumina NextSeq 500. The panel covered 63 genes described to play a role in DNA repair or treatment resistance, including *ABCB1* and *TP53*. Sequencing data were aligned to the Genome Reference Consortium human genome assembly (GRCh37 b37) using BWA mem (v0.7.17-r1188). Variants were called using 4 tools: Mutect2 (v4.0.11.0), VarDictJava (v1.5.7), Strelka2 (v2.9.9) and Varscan2 (v2.4.3) and were annotated using the Ensembl Variant Effect Predictor (v92.4). Those variants identified by a single caller were not analyzed further, and variants with a combined variant allele frequency of <10% across all lines were also discarded. The *TP53* mutations were manually reviewed in Integrative Genomics Viewer (IGV).

### 2.11. Statistical Analysis

All statistical analyses were performed using GraphPad Prism software (Version 9.0.2) or R (Version 3.6.1). Data are expressed as mean ± standard error of the mean (SEM) as indicated, from a minimum of 3 independent measurements. The minimum threshold for rejecting the null hypothesis was *p* < 0.05.

## 3. Results

### 3.1. SLC25A40-ABCB1 Fusions Mediate Multi-Drug Resistance, Decreased Proliferation and Elevated Oxidative Metabolism

In order to decipher the biological consequences of *SLC25A40-ABCB1* fusions beyond drug resistance, we utilized our AOCS18.5 HGSOC patient-derived cell line (parental) [13] which contains approximately 40% fusion-positive cells. Single-cell cloning of this parental line generated five *SLC25A40-ABCB1* fusion-negative (Clone B, C, D, E and F) and five fusion-positive (Clones 8, 9, 13, 15B and 18B) clones. The *SLC2540-ABCB1* DNA breakpoint was only detected in the five fusion-positive clones, with complete absence in ovarian control cell lines (JHOS2, SKOV3 and AOCS21.2) (Figure S1A). As a result, total *ABCB1* (mRNA and MDR1 protein) and *SLC25A40-ABCB1* (mRNA) expression levels were significantly higher in fusion-positive clones (*p* < 0.0000 and *p* < 0.0001, respectively) with *ABCB1* mRNA levels strongly correlating with *SLC25A40-ABCB1* expression (*R*^2^ = 0.9739) (Figure S1B–D).

**Figure 1:**
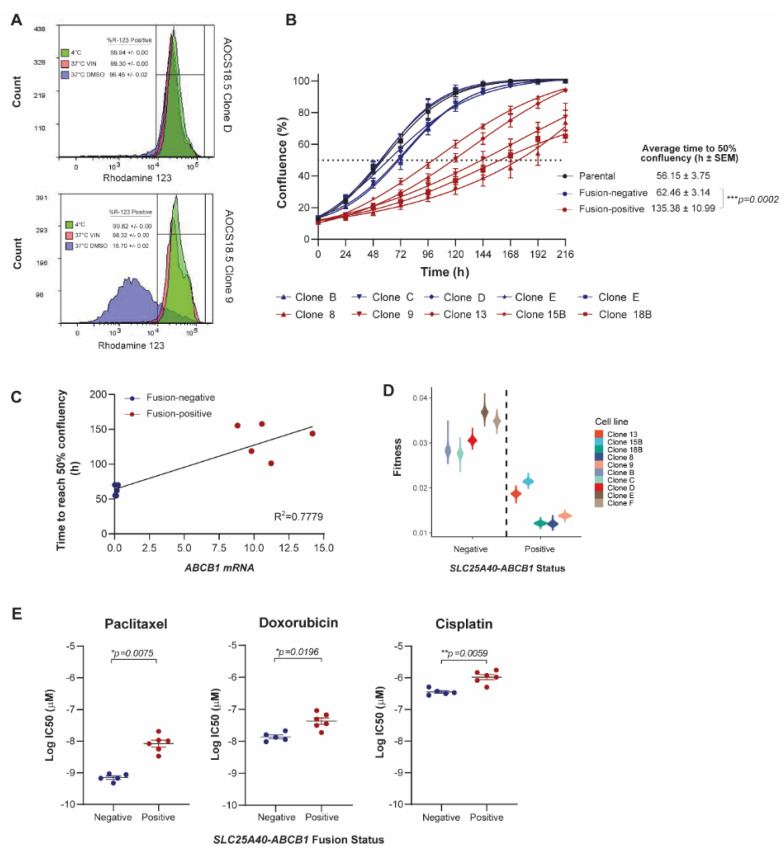

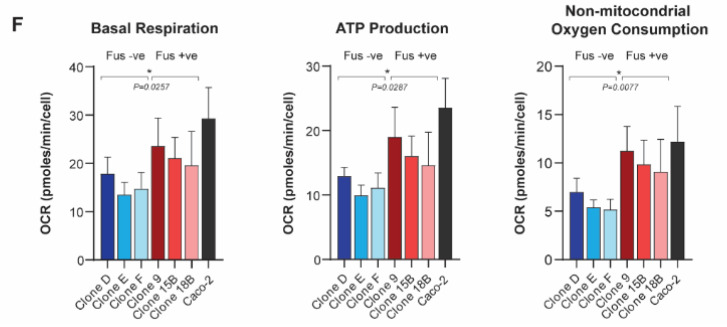
*SLC25A40-ABCB1* fusions impair the proliferative fitness of HGSOC cells and mediate changes in drug sensitivity and metabolism. (**A**) Representative flow cytometric analysis of rhodamine 123 efflux via P-gp in AOCS18.5 *SLC25A40-ABCB1* fusion-negative (Clone D) and fusion-positive (Clone 9) clones. Treatments are as follows: 4 °C (green, P-gp inactive), 37 °C with *ABCB1* competitive substrate Vinblastine (VIN) (pink), and 37 °C DMSO (pink, P-gp active). (**B**) IncuCyte proliferation analysis of AOCS18.5 parental (black), fusion-negative (blue) and fusion-positive (red) clones over 216 h. Dashed line denotes 50% confluency. (**C**) Correlation between total *ABCB1* mRNA expression and time to reach 50% confluency in fusion-negative (blue) and fusion-positive (red) clones. (**D**) Absolute fitness of fusion-negative and positive clones according to intrinsic growth rate. (**E**) Relative viability (IC_50_) of fusion-negative and positive cells following 72 h treatment with P-gp substrate (doxorubicin, paclitaxel) and non-substrate (cisplatin) therapy. Viability assessed via DAPI staining. (**F**) Basal respiration, ATP production and non-mitochondrial oxygen consumption rate of fusion-negative and positive clones evaluated through oxygen consumption rate (OCR) analysis (Seahorse extracellular flux assay). Data normalized to cell number. Caco-2, a known *ABCB1* overexpressing colon carcinoma cell line (*SLC25A40-ABCB1* fusion-negative). Data represents mean ± SEM from a minimum of 3 independent experiments. Asterisks denote statistical significance * *p* < 0.05, ** p < 0.01, *** p < 0.001.

To assess the consequence of *SLC25A40-ABCB1* fusions on P-gp activity, efflux of the highly specific *ABCB1* substrate rhodamine was assessed in fusion-positive and fusion-negative clones. Following 37 °C treatment, cellular retention of rhodamine was significantly higher in fusion-negative clones (98.20% ± 0.01 versus 14.94% ± 0.01, *p* < 0.001), with AOCS18.5 fusion-positive clone efflux levels similar to those observed in the known *SLC25A40-ABCB1* fusion-negative cell line Caco-2, which highly expresses *ABCB1* (75.59% efflux) (Figure 1A and Figure S2).

The synthesis and maintenance of ATP-dependent ABC pumps on the apical cell membrane and transport of substrates requires a considerable investment of energy [26]. For cancer cells, this metabolic cost is observed even in the absence of drugs and requires a significant diversion of resources that would ordinarily be devoted to processes such as invasion or proliferation [27]. IncuCyte live-cell proliferation assays were undertaken to assess whether the presence of *SLC25A40-ABCB1* fusions alters the proliferative fitness of HGSOC cells. Indeed, fusion-positive AOCS18.5 clones required 2.18-fold more time to reach 50% confluency compared to their fusion-negative counterparts (average 62.46 ± 3.14 versus 135.38 ± 10.99 h, respectively) (*p* = 0.0002) with *ABCB1* mRNA expression levels strongly correlating with cellular proliferative times (*R*^2^ = 0.7779) (Figure 1B,C and Table S1).

To further examine whether *SLC25A40-ABCB1* fusions modulate cellular fitness, the overall fitness of fusion-negative and positive clones was compared. A logistic population growth model was fitted to each clone’s 2D growth curves and the estimated intrinsic growth rate parameter provided an absolute fitness measure of each clone. The fitness of fusion-negative clones was 2-fold greater (1.9–2.3) than fusion-positive clones (df = 8, t = −6.1, *p* < 0.0005) (Figure 1D). Indeed, the population doubling time for fusion-negative clones was 22 h versus 44 h for fusion-positive clones, highlighting that *ABCB1* fusions significantly impair the proliferative fitness of HGSOC cells.

As overexpression of *ABCB1* is one of the key factors leading to cancer MDR, we next sought to solidify the association of *SLC25A40-ABCB1* fusions with resistance to standard of care HGSOC chemotherapeutic agents. AOCS18.5 parental cells, as well as fusion-negative (*n* = 5) and fusion-positive clones (*n* = 5), were treated with P-gp substrate (doxorubicin, paclitaxel) and non-substrate (cisplatin) agents for 72 h, and their viability was determined through DAPI staining. As expected, compared to fusion-negative clones, fusion-positive clones were 3.8 and 15.2-fold more resistant to doxorubicin and paclitaxel, respectively (doxorubicin IC_50_ 14.48 ± 2.05 versus 55.26 ± 11.19 nM, *p* = 0.0071; paclitaxel IC_50_ 0.72 ± 0.08 versus 11.05 ± 2.52 nM, *p* = 0.0035). Fusion-positive clones were also 3.54-fold more resistant to the non-P-gp substrate cisplatin (IC_50_ 379.95 ± 38.74 versus 1343.61 ± 185.58 nM, *p* = 0.0009) (Figure 1E) (Figure S3A). Indeed, cisplatin sensitivity was strongly correlated with proliferative rate (*R*^2^ = 0.878) with slower growing fusion-positive lines (>100 h to reach 50% confluency) requiring significantly higher levels of cisplatin to achieve an IC_50_ (*p* = 0.0003) (Figure S3B).

Finally, as substrate transport mediated by P-gp is ATP-dependent (P-gp hydrolyzes two ATP for every extruded molecule) [28], we next investigated whether *ABCB1* fusions mediate basal changes in mitochondrial respiration. Seahorse Mito Stress Test assays were employed to assess seven parameters of energy expenditure in fusion-negative and positive cells. Of these markers, basal respiration (*p* = 0.026), ATP production (*p* = 0.029) and non-mitochondrial oxygen consumption (*p* = 0.008) were significantly elevated (1.39–1.72 fold) in fusion-positive lines, suggesting that presence of the fusion causes elevated basal oxidative metabolism to compensate for the increased energy demand (Figure 1F) (Figure S3C). Together, our results show that in addition to driving drug resistance, *SLC25A40-ABCB1* fusions result in proliferative and metabolic fitness penalties.

### 3.2. SLC25A40-ABCB1 Fusions Mediate an ECM/Inflammatory Enriched Transcriptional Profile

To investigate whether additional genomic changes are associated with the acquisition of *SLC25A40-ABCB1* fusions, targeted sequencing of 63 genes involved in DNA repair or chemotherapy resistance in HGSOC was performed on fusion-negative (*n* = 5) and positive clones (*n* = 5). The parental line and all single-cell clones contained the p.L132M *TP53* driver mutation (VAF > 0.99). No mutations were found exclusively in all fusion-positive clones (Figure S4).

**Figure 2:**
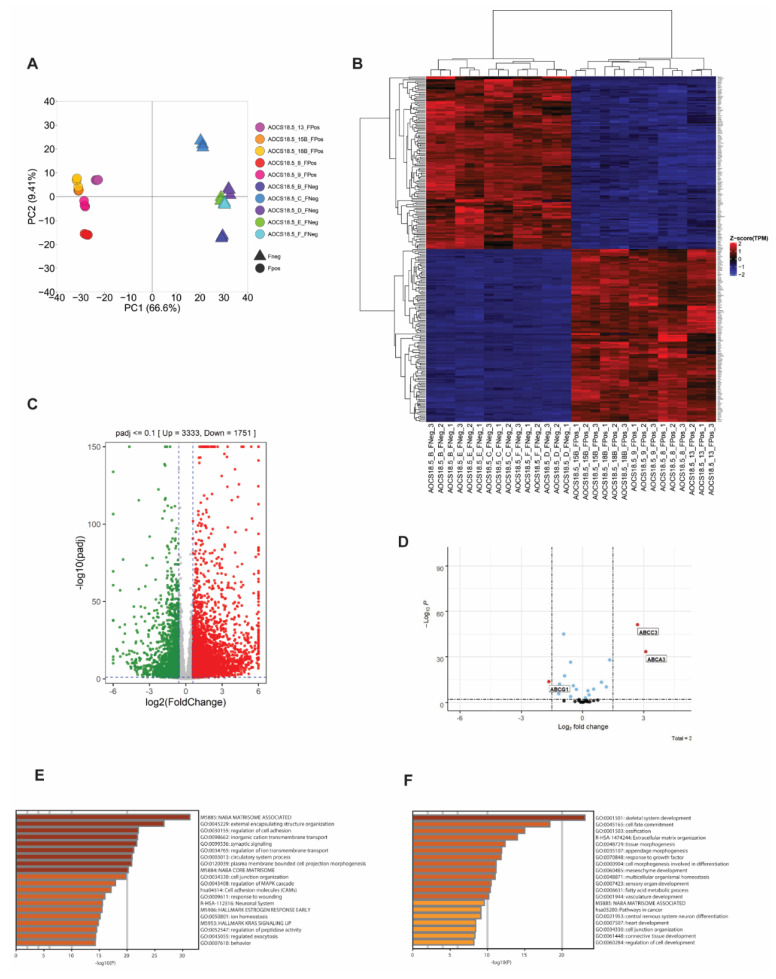
*SLC25A40-ABCB1* fusions induce strong enrichment for matrisome-associated genes. (**A**) Principal Component Analysis (PCA) of gene expression data for *ABCB1* fusion-negative (5 clones, triangle) and fusion-positive (5 clones, circle) cell lines. (**B**) Unsupervised hierarchical clustering analysis of the transcriptional profile of *SLC25A40-ABCB1* fusion-negative (FNeg, *n* = 5) and fusion-positive (FPos, *n* = 5) AOCS18.5 single-cell clones. The top 250 significantly differentially expressed genes from RNAseq analysis are shown (*p*-adj <0.1). (**C**) Volcano plot showing significantly up/downregulated differentially expressed genes in fusion-positive clones compared to fusion-negative. Cut-off criteria was log_2_fold change of 1.5 and *p*-adj of <0.1. (**D**) Identification of ABC family members modulated by *SLC25A40-ABCB1* fusions in addition to *ABCB1*. Cut-off criteria was log_2_ fold change of 1.5 and *p*-adj of <0.1. Metascape analysis of pathways enriched (**E**) and repressed (**F**) in AOCS18.5 fusion-positive clones.

Besides direct upregulation of *ABCB1* as a consequence of *SLC25A40-ABCB1* fusions, it is unknown whether these fusions impart additional baseline transcriptional changes that could account for the phenotypic differences observed. As such, RNAseq analysis of five fusion-negative (Clones B, C, D, E, F) and five fusion-positive (Clones 8, 9, 13, 15B, 18B) AOCS18.5 clones was conducted. Principal component analysis (PCA) of these transcriptomes demonstrated that *SLC25A40-ABCB1* fusion-positive lines shared similar basal gene expression profiles and clustered separately from fusion-negative clones (Figure 2A). In all, 3333 genes were significantly upregulated (>1.5 log_2_ fold change, *p*-adj < 0.1) in fusion-positive lines, with 1751 genes significantly downregulated (Figure 2B,C). The top five significantly upregulated genes included *AJAP1* (11.34-fold increase), *KLK11* (11.31-fold), *DCAF4L2* (11.01-fold), *EREG* (10.56-fold), and *WBSCR17* (9.80-fold). Using a stringent cut-off of >2.0-fold change (*n* = 2238 differentially expressed genes), Metascape pathway analysis [29] revealed that the most significantly enriched pathways in fusion-positive clones included the NABA matrisome-associated pathway (160/751 genes, 7.24%), followed by external encapsulating structure organization (102/398 genes, 4.62%) and regulation of cell adhesion (139/734 genes, 6.29%) (Figure 2E). The core matrisome pathway comprises 274 genes primarily encoding extracellular matrix (ECM) glycoproteins, collagens and proteoglycans which provide multiple inputs governing cell survival, proliferation, differentiation, shape, polarity and motility [30,31].

In contrast, the top five significantly upregulated genes in fusion-negative lines included *MAGEB2* (11.97-fold increase), *SOX11* (8.68-fold), *TBC1D10C* (8.29-fold), *FMOD* (7.32-fold), and *MAGEA3* (7.30-fold). *MAGEB2* belongs to the melanoma antigen gene (MAGE-I) family of cancer testis antigens whose expression is restricted to normal testis but are aberrantly expressed in a broad number of human tumors. *MAGEB2* has been shown to promote tumor cell proliferation in a p53-independent fashion and enhances E2F transcriptional activity and resistance to ribotoxic stress [32,33]. Metascape pathway analysis (≥2.0-fold cut-off, *n* = 842 genes) showed the top enriched pathways included skeletal system development (64/486, 7.71%), cell-fate commitment (41/251, 4.94%) and ossification (47/402, 5.66%) (Figure 2F).

TRRUST (transcriptional regulatory relationships unraveled by sentence-based text-mining) analysis for transcriptional regulatory relationships [34] revealed significant enrichment for genes primarily regulated by *SP1* (*n* = 99 genes) and by *NKFKB1* (*n* = 65 genes) in fusion-positive lines (Figure S5A). The proximal promoter of *ABCB1* contains several regulatory regions, including a GC rich element (position −56 to −42) required for constitutive promoter activity. Interestingly, several studies have shown that the transcriptional activator SP1 is the predominant factor that binds to this GC box within the *ABCB1* promoter, mediating its activation in the absence and presence of genotoxic stress [35,36].

In addition to *ABCB1*, at least 16 other ABC transporters from 4 ABC subfamilies have been implicated in the transport of chemotherapeutic agents and/or in mediating MDR [12,37]. We therefore next investigated whether any other ABC family members are differentially expressed in fusion-positive cell lines. Out of the 35 ABC members examined, in addition to *ABCB1*, 2 ABC family members were significantly upregulated: *ABCA3* and *ABCC3*, with *ABCG1* significantly repressed (3.11, 2.71 and −1.65 log_2_ fold change respectively) (*p* < 0.0001) (Figure 2D). Both *ABCA3* and *ABCC3* have well-established roles in the efflux of anthracycline and methotrexate/epipodophyllotoxin chemotherapy substrates, respectively [38], with several studies reporting *ABCA3* (known for its role in the production of pulmonary surfactant) to be upregulated in cisplatin-resistant ovarian cancer cells [15].

To extend our cell line transcriptional profile findings, we evaluated the expression profiles of our previously characterized cohort of *SLC25A40-ABCB1* fusion-negative (*n* = 21) and fusion-positive (*n* = 6) HGSOC patient ascites samples [7,13]. Similar to our cell line analysis, unsupervised hierarchical clustering of these transcriptomes demonstrated that *SLC25A40-ABCB1* fusion-positive tumors share similar basal gene expression profiles and distinctively cluster from fusion-negative tumors (Figure S5B). In all, 100 genes were significantly upregulated (>1.5log_2_ fold change, *p*-adj < 0.1) in fusion-positive samples, with 162 genes significantly downregulated. The top five significantly upregulated genes included *SERPINB7* (6.01-fold increase), *LBP* (5.96-fold), *BHMT2* (5.63-fold), *ORM1* (5.40-fold) and *NOS1* (4.93-fold), with the top five downregulated genes being *SAGE1* (24.98 decrease), *CEACAM5* (6.47-fold), *TMTM179* (6.25-fold), *FGB* (6.10-fold) and *CPB1* (6.05-fold) (Figure S5C). Metascape pathway analysis of upregulated genes demonstrated enrichment for response to bacterium (16/728, 16%), folate metabolism (6/73, 6%), intrinsic apoptotic signaling (8/283, 8%) and NABA matrisome (12/751, 12%) (Figure S5D).

**Figure 3:**
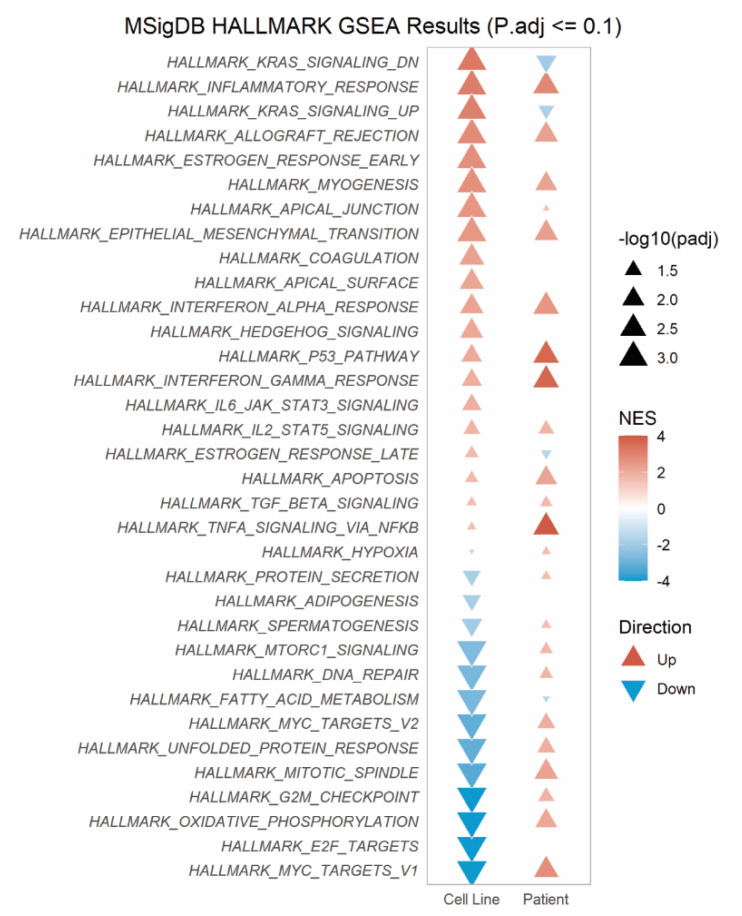
*SLC25A40-ABCB1* fusions induce strong enrichment for inflammatory response pathways across fusion-positive cell lines and relapsed HGSOC patient samples. MSigDB Gene Set Enrichment (GSEA) using fGSEA on Hallmark pathways enriched in AOCS18.5 *SLC25A40-ABCB1* fusion-positive clones and relapsed HGSOC ascites samples. Normalized enrichment score (NES) and significance shown.

Gene set enrichment analysis (GSEA) of 34 Hallmark pathways revealed 12 pathways significantly enriched across both the cell line and patient RNAseq data sets (Figure 3). These pathways primarily concerned inflammatory response (inflammatory, allograft rejection, interferon alpha/gamma response pathways), myogenesis and epithelial-to-mesenchymal transition (EMT). In contrast, only one pathway, fatty acid metabolism, was consistently downregulated across both cohorts. Together, our findings suggest that in addition to the direct upregulation of *ABCB1*, *SLC25A40-ABCB1*, fusions result in ECM and inflammatory-enriched transcriptional profiles.

### 3.3. SLC25A40-ABCB1 Fusion-Positive Cells Display Altered Cellular Morphology

**Figure 4:**
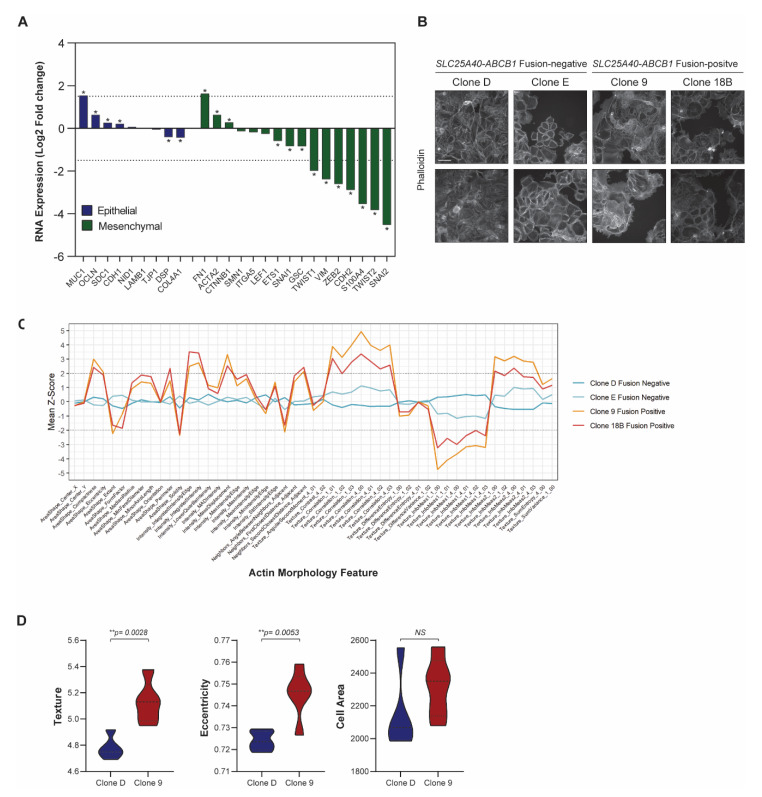
*SLC25A40-ABCB1* fusion-positive cells display strong downregulation of mesenchymal genes. (**A**) RNA expression of well-established epithelial (*n* = 9, blue) and mesenchymal markers (*n* = 16, green) in *SLC25A40-ABCB1* fusion-positive clones relative to their fusion-negative counterparts. Asterisks denote statistical significance * *p* < 0.05. (**B**) Representative phalloidin staining of F-actin filaments in AOCS18.5 fusion-negative (Clone D, E) and fusion-positive (Clone 9, 18B) clones. Pictures taken at 10× magnification with 100 µM scale bar shown. (**C**) Phenotypic profiles showing 51 actin morphology features across AOCS18.5 fusion-negative and positive clones. Values were normalized by fold changing to the median of Clone D and Clone E cells (baseline). Z-Scores reflect the difference between a particular cell line and the median of clone D and E. (**D**) IncuCyte cell-by-cell analysis comparing texture, area and eccentricity of *SLC25A40-ABCB1* fusion-negative (blue) and fusion-positive (red) AOCS18.5 clones. Data represents mean feature ± SEM from 4 independent replicates (500 cells minimum). Asterisks denote statistical significance ** p < 0.01.

As the presence of *SLC25A40-ABCB1* fusions drove a clear ECM/EMT transcriptional profile, we next investigated the morphological consequences of *ABCB1* fusions in the cell lines. There was a significant downregulation of 10/16 (62.5%) well-established mesenchymal genes [39] in fusion-positive cell lines, with the zinc finger protein *SNAI2* (4.52 log_2_ fold, *p*-adj < 0.0001) as the most repressed target (Figure 4A). In contrast, 4/9 (44.4%) epithelial marker genes [39] were significantly upregulated in fusion-positive clones, with *MUC1* (type-I transmembrane glycoprotein) showing the greatest induction (1.54 log_2_ fold, *p*-adj < 0.0001). *MUC1* has been shown to modulate cell–cell and cell–extracellular matrix interactions by steric hindrance, and its overexpression has been suggested to promote metastasis through disruption of these interactions [40].

Phalloidin staining of F-actin filaments was conducted in AOCS18.5 fusion-negative (Clone D, Clone E) and fusion-positive (Clone 9 and Clone 18B) cell lines to assess phenotypic differences (general intensity, texture and shape features) (Figure 4B). Of the actin features assessed, 45/51 (88.2%) were significantly different (*p* < 0.05) (Figure 4C). The top 4 features significantly modulated by *ABCB1* fusion status were solidity (*p* = 1.076 × 10^−20^), texture correlation (*p* = 7.907 × 10^−20^), mass displacement (*p* = 2.727 × 10^−19^) and compactness (*p* = 1.511 × 10^−18^). Furthermore, IncuCyte cell-to-cell analysis revealed that although cell area was not significantly divergent, cellular texture (*p* = 0.0028) and eccentricity (*p* = 0.0053) was altered in fusion-positive lines (Figure 4D). Together, these data suggest that in comparison to *ABCB1* fusion-negative cells, drug-resistant fusion-positive cells may possess an epithelial state with stronger cell–cell adhesions and limited migratory potential.

### 3.4. Clonal Co-Operation between Fusion-Negative and Positive Cells Does Not Promote Proliferative Fitness

**Figure 5:**
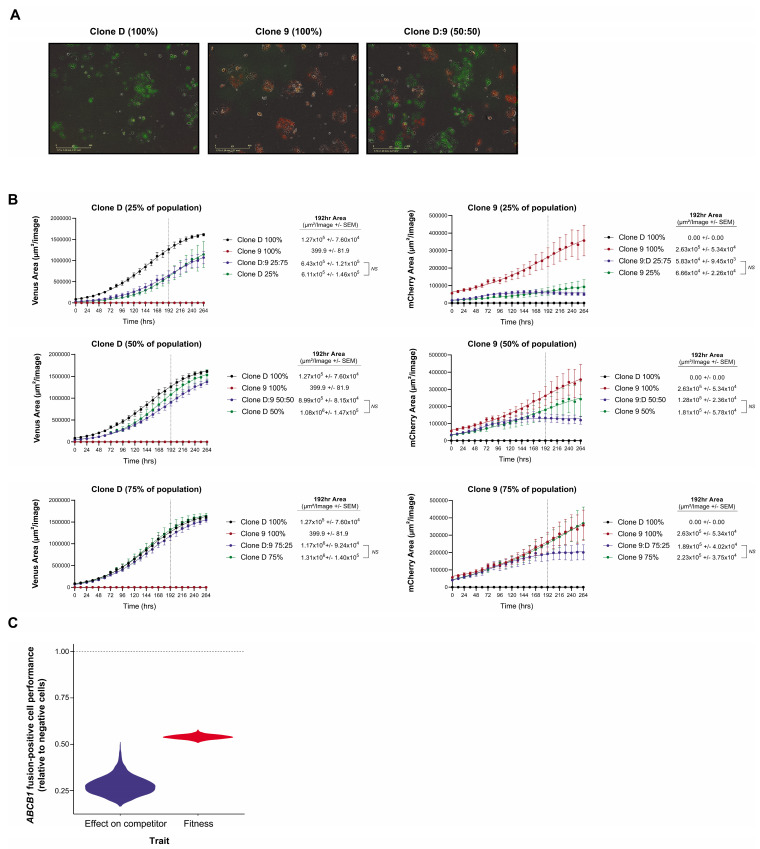
Competition model estimates of the relative fitness and competitive ability of fusion-positive versus negative counterparts. (**A**) Representative IncuCyte fluorescent images of Clone D (Venus Yellow) and Clone 9 (mCherry) LeGO transduced AOCS18.5 clones seeded in pure (100%) and mixed (50:50) ratios 48 h post seeding. (**B**) IncuCyte proliferation analysis of Clone D (Venus Yellow) and Clone 9 (mCherry) cells grown alone or in co-culture (25:75, 50:50 and 75:25 seeding ratios) for 264 h. Data represent mean fluorescent area (µM^2^/image) ± SEM from a minimum of 4 independent experiments. (**C**) Estimates obtained by fitting a Lotka–Volterra population growth model of competition to the growth curves of fusion-positive (Clone 9) and fusion-negative (Clone D) clones’ growth in mono- and co-culture. Violin distributions show the range of uncertainty in the relative performance of the resistant and sensitive cells. Values below the horizontal dashed line indicate a greater fitness and competitive effect of the sensitive *ABCB1* fusion-negative clone.

As we had previously demonstrated that *SLC25A40-ABCB1* fusions are frequently subclonal [7,13] and that they endow a proliferative fitness penalty (Figure 1B), we next investigated which population would outcompete the other when co-cultured in the absence of selective pressure to recapitulate clinical settings when patients are off treatment. Fusion-negative (Clone D) and fusion-positive (Clone 9) clones were transduced with lentiviral gene ontology (LeGO) vectors encoding either Venus Yellow or mCherry, seeded in a 50:50 ratio and co-cultured (Figure 5A). As expected, fourteen days post seeding, FACS analysis revealed that 99.38% ± 0.069 of the cellular population was enriched for fusion-negative cells. Similarly, one month post co-culture only 0.02% ± 0.01 of the cellular population contained fusion-positive cells, with *SLC25A40-ABCB1* mRNA expression undetectable (Figure S6A–C).

To extend these findings we next determined whether potential cross-talk between fusion-negative and positive cells impedes or enhances the proliferative capacity of either population. Clone D and Clone 9 LeGO-labelled cells were seeded either alone or in three mixed ratios (25:75%, 50:50% and 75:25%) and grown for 11 days. Interestingly, IncuCyte proliferation analysis revealed no significant difference in proliferative capacity (intrinsic population growth rate) when cells were co-cultured for all ratios, possibly suggesting that drug-sensitive and drug-resistant cells do not co-operate to modulate the proliferative fitness of each other (Figure 5B). However, at higher densities, resistant cells are outcompeted by sensitive cells, whilst sensitive cell abundances are barely impacted by the presence of the resistant competitors. The fitted Lotka–Volterra model of population growth and competition estimated the competitive effect of sensitive cells on resistant cells to be 3.5 times as great as the competitive effects that resistant cells had on them (Figure 5C).

Finally, to examine how clonal composition influences chemosensitivity, 72 h drug sensitivity assays (doxorubicin, cisplatin and paclitaxel) were conducted with fusion-negative (Clone D and Clone E) and fusion-positive (Clone 9 and Clone 18B) clones seeded alone or in mixed ratios (25:75%, 50:50% and 75:25%). Most surprisingly, no significant differences in IC_50_ values were observed in the co-culture assays when compared to 100% fusion-negative controls, with one exception in paclitaxel treated cells at 25:75% ratio (Table 1) (Figure S7). For example, no significant difference in IC_50_ values was demonstrated when doxorubicin assays were performed on mixed cellular populations containing 75% fusion-positive Clone 18B/25% fusion-negative Clone E cells, versus 100% Clone E fusion-negative cells (IC_50_ 46.33 ± 6.60 versus 43.53 ± 6.878 respectively). These findings highlight the importance of chemotherapeutic protocols, which maintain mixed clonal states to persevere drug sensitivity instead of regimens which drive the whole tumor population towards drug resistance.

### 3.5. High-Throughput Drug Screening Identifies FDA Agents That Induce Cytotoxic Responses Regardless of Fusion Status and Clonal Composition

To date, there remains no standard of care for patients whose tumors harbor *ABCB1* fusion-mediated drug-resistant cancer cells. As the original AOCS18.5 patient-derived cell line contained a mixture of both *ABCB1* fusion-negative and positive cells, our next goal was to identify rapidly translatable FDA-approved agents that fusion-negative/positive cells were equally susceptible to. High-throughput screening of 2907 FDA-approved compounds was performed in two AOCS18.5 fusion-negative lines (Clone D and Clone E), two fusion-positive lines (Clone 9 and Clone 18B) and a Clone D/Clone 9 (50:50 mix) to recapitulate a mixed population state. Primary screening identified 103 compounds in which normalized cell counts were <50% from control (DMSO) at a minimum of one dose point (0.05, 0.5, 5 µM) in all four cell lines (Table S2). In all, 36/103 (35.0%) compounds reduced viability across 2–3 dose points and included agents such as vincristine, topotecan, docetaxel and camptothecin. Using a viability Z-score criteria < −2 across a minimum of two dose points, 36 agents were nominated for further validation. Confirmatory assays were conducted using a six-point dose curve (0.01–10 µM) and 72 h of treatment.

As cellular division rate is a powerful confounder in the calculation of IC_50_ values, the normalized growth rate inhibition (GR) method was used to correct for variation in division rates by estimating the magnitude of drug response on a per division basis [41]. Using this metric, no significant difference in GR_50_ values (the concentration at which the effect reaches a growth rate (GR) value of 0.5) was observed between the *ABCB1* fusion-negative and fusion-positive lines for 35/36 (97.2%) agents (Table 2) (Figure S8). Of the 36 compounds, the DNA synthesis inhibitor bleomycin was the only agent identified in which GR_50_ values were significantly different between fusion-negative and fusion-positive lines (0.258 µM versus 0.529 µM respectively) (*p* = 0.028). Due to high potency (>80% reduction in cell viability) at the lowest compound dose tested (0.01 µM), GR_50_ were unattainable for three agents (colchicine, docetaxel and podofilox) (Table 2). Indeed, the top five most potent (GR_50_ < 0.02 µM) FDA-approved agents included mitoxanthrone, proscillaridin, triptolide, vinblastine and convallatoxin. Furthermore, regardless of fusion status, cell cycle profiles were consistent across cell lines with strong G0-G1 cell cycle arrest induced by 63.9% (23/36, fraction range 26.9–58.1%, 0.5 and 2.25 µM) of compounds followed by above G2-M arrest (11/36 compounds, fraction range 27.9–44.4%, 0.5 and 2.25 µM) (Figure S9).

As the selective pressure of chemotherapy will drive the clonal evolutionary development of both drug-sensitive and drug-resistant populations within the same tumor, we next examined whether agents identified from our primary screen were equally cytotoxic for pure *ABCB1* fusion-negative (Clone D) and mixed-fusion (Clone D and Clone 9, 50:50 mix) populations. Interestingly, no significant differences in GR_50_ values were observed between the two populations for any of our 36 compounds (Table 2) (Figure S8), highlighting that our identified FDA agents could provide new therapeutic avenues for relapsed HGSOC patients that harbor pure or mixed tumor populations.

## 4. Discussion

Cancer is a highly complex, adaptive system that can rapidly evolve new phenotypic and genotypic profiles to circumvent therapy. Indeed, the emergence of drug resistance remains our largest impediment in the quest towards curative cancer treatment, with an estimated 90% of all cancer related deaths attributed to chemoresistance [42]. Although the basis of drug resistance is multifactorial involving both tumor- and drug-related factors, overexpression of *ABCB1* is the most common phenomenon employed by cancer cells to diminish the intracellular accumulation of chemotherapeutic agents, with approximately 50% of all anticancer agents used in the clinic effluxed by this transporter [43]. It is well established that high ABC transporter expression is associated with worse survival outcomes [44]. Indeed, several studies, including a meta-analysis of 38 retrospective studies assessing 8067 epithelial ovarian cancer cases, demonstrated that *ABCB1* over-expression was a significant risk factor associated with unfavorable overall and progression-free survival [45,46]. As it has become increasingly apparent that MDR is a multifactorial phenomenon, we need to shift our focus from the development of targeted ABCB1 agents to therapeutic strategies that can disrupt ABCB1-mediated MDR-contributing factors.

We show that in addition to driving drug resistance, *ABCB1* fusions significantly reduced the proliferative fitness of HGSOC cells in the absence of the selective pressure of chemotherapy. We hypothesize that this is primarily due to the higher bioenergetic demand of fusion-positive cells, as evidenced by elevated basal respiration, necessary for the movement of endogenous molecules such as cholesterol, phospholipids and sphingolipids. Few studies have focused on the role of ABCB1-associated malignant proliferation. The stable knockdown of *Mdr1a/1b* in mouse colon carcinoma cells significantly inhibited cellular growth both in vitro and in vivo [47]. Similarly, the suppression of intestinal tumorigenesis (small intestinal polyps) was observed in *Mdr1a/b* knockout mice (APC mutant background) [48]. Interestingly, *Mdr1a/1b*^−/−^ mice are viable and show no functional deficiency in terms of fertility, and abnormalities across a range of histological, haematological, serum–chemical, and immunological parameters [49]. The clear lack of human-based *ABCB1* knockout studies warrants further investigations into *ABCB1*’s role in the regulation of cellular proliferation and how this “fitness” cost can be exploited therapeutically.

The expenditure of energy required to maintain the molecular machinery that governs MDR phenotypes is not evolutionarily favored except when chemotherapy is administered. In this case, the energy demands required to drive ABCB1’s drug export function increases survival and therefore confers increased fitness. However, in the absence of chemotherapy, the cost of ABC pumps serves no survival benefit and therefore reduces fitness due to the added energetic cost. For many years, clinical practice has been dominated by the concept of the maximum tolerated dose (MTD), which drives the development and evolution of drug resistance through the intense selection pressures imposed by cytotoxic therapies. This strategy thereby essentially accelerates the proliferation of drug-resistant cells by selecting for resistant clones and eliminating all competing drug-sensitive populations [50,51]. We have clearly demonstrated the proliferative deficiencies of drug-resistant *ABCB1* fusion-positive cells, and this can potentially be exploited through adaptive therapy to inhibit population expansion. Adaptive therapy capitalizes on the competitive interactions between drug -sensitive and drug-resistant subclones to maintain a controllable stable cancer population below a certain symptomatic threshold whilst maintaining a substantial population of treatment-sensitive cells [1]. As growing evidence suggests that cancer cells behave as communities, by manipulating Darwinian evolutionary principles, this approach aims to further suppress the proliferation of less fit resistant populations. Indeed, our clonal mixture drug assays demonstrated that even if tumor populations contain as little as 25% *ABCB1* fusion-negative drug-sensitive cells (75% fusion-positive), chemotherapeutic IC_50_ values (doxorubicin, paclitaxel and cisplatin) were virtually the same as 100% fusion-negative alone, underscoring the importance of maintaining drug-sensitive populations to outcompete and compete with resistant communities during off-treatment periods. Finally, our high-throughput drug screening efforts importantly identified 36 FDA compounds that exert equal cytotoxicity in fusion-negative and positive clones (pure and mixed populations). Further investigations into agents whose primary mode of action is not dependent on cell cycle progression rates (e.g., inhibitors of microtubule polymerization) are warranted. In particular, enrichment for agents that target Na^+^/K^+^-ATPase (e.g., digitoxin, convallatoxin) and DNA topoisomerase I (e.g., camptothecin, irinotecan) was observed.

One of the striking findings revealed from our study was the clear enrichment for genes associated with the matrisome (ECM markers) and EMT in fusion-positive cells. This may be the driving mechanism for their observed altered actin feature morphology (compactness, eccentricity and texture etc.). Our RNA-seq analysis revealed the putative tumor suppressor *AJAP1* as the most highly expressed gene in fusion-positive lines. AJAP1 is a type-1 transmembrane protein that localizes and interacts with the E-cadherin-catenin complex and is involved in cellular processes such as cell migration and invasion by modulating adherens junctions and remodeling the ECM and cytoskeleton [52]. For example, in nonpolarized, highly migratory and invasive cells, AJAP1 interacts with the transmembrane glycoprotein CD147, an invasion-promoting protein [53]. The stable overexpression of *AJAP1* in MCF7 breast cancer cells accelerated cell migration with knockdown decreasing migratory behavior [54]. Paradoxically, the overexpression of *AJAP1*-attenuated glioblastoma cell line adhesion capacity to extracellular matrix components (laminin, collagen IV and fibronectin), with delayed wound-healing closure only observed on fibronectin-coated plates [55]. Concordant with this study, the knockdown of *AJAP1* enhanced the migratory and invasive behavior of primary endothelial HUVEC cells [52].

Cancer invasiveness has long been associated with increased drug resistance, yet little is known about the molecular mechanisms linking these two processes. Indeed, the highly conserved cellular process of EMT has emerged as a major contributor to therapy resistance by permitting polarized, immobile epithelial cells to transform into mesenchymal mobile cells due to loss of apico-basal polarity and cell–cell contacts [56].We observed strong repression of several mesenchymal genes in fusion-positive lines including *SNAI1 (SNAIL)*, *SNAI2 (SLUG)*, *Vimentin (VIM)* and *TWIST*. Interestingly, a bioinformatic-based analysis of the promoter regions of 16 ABC transporters by Saxena et al. revealed binding sites for several EMT-inducing transcription factors including *SNAI1*, *SNAI2*, *TWIST*, *E12*, *E47* and *FOXC2*, with *TWIST*, *SNAI1*, and *FOXC2* capable of increasing the promoter activity of ABC transporters [57]. However, it must be noted that the fused transcript identified in AOCS18.5 cells is a result of a 250 kb intergenic deletion, fusing the promoter and non-coding exon 1 of *SLC25A40* to exon 2 of *ABCB1* [7]; hence EMT transcription factor ABCB1 promoter sites are absent.

Although direct evidence linking ABC transporters and metastasis is limited, roles are emerging for these proteins in cell migration and invasion. Attenuation of *ABCB1* reduced the migratory capacity of both MCF-7 breast carcinoma and rat brain endothelial cells, with overexpression associated with increased migration [58,59]. Increased migratory ability of MCF-7-ADR-1024 doxorubicin-resistant cells was also exhibited compared to wildtype control lines [60]. Finally, chemotactic response and migration of peripheral dendritic cells to lymph nodes is significantly reduced in *Abcc1*^−/−^ mice [61]. Although we demonstrated strong transcriptional repression of mesenchymal programming, it is unknown whether patients whose tumors contain fusion-positive cells exhibit a greater degree of metastatic disease or whether these cells have a proclivity to remain within the ascites peritoneal fluid.

## 5. Conclusions

ABC transporters have a pivotal role in host cell detoxification and protection of the body against xenobiotics. However, a considerable body of evidence also points to their fundamental roles in tumor biology. This is the first study to provide significant insights into the phenotypic, metabolomic and transcriptional consequences of *SLC25A40-ABCB1* fusions beyond drug resistance. We show that during treatment “holidays”, the proliferative fitness deficits of fusion-positive cells will allow fusion-negative cells to outcompete drug-resistant populations. More importantly, we have identified FDA-approved agents that induce equal cellular cytotoxicity regardless of fusion status. Taken together, our findings will have significant implications in guiding changes to current HGSOC treatment regimens, facilitating our ultimate goal of long-term cancer control. From a translational perspective, steering the evolutionary dynamics of *ABCB1* acquired resistance through the inhibition of metabolism or ECM may represent a novel therapeutic approach for ABCB1-mediated acquired resistance in HGSOC patients as well as for all *ABCB1*-driven MDR cancers.

## Figures and Tables

**Table 1:** Influences of clonal composition in mediating chemosensitivity.

**Cisplatin**	**Clone D (Fus − ve) and Clone 15B (Fus + ve)**			**Clone E (Fus − ve) and Clone 9 (Fus + ve)**			**Clone E (Fus − ve) and Clone 18B (Fus + ve)**		
	**72 h IC_50_ (µM)**	**^ *p*-Value**	**^#^ *p*-Value**	**72 h IC_50_ (µM)**	**^ *p*-Value**	**^#^ *p*-Value**	**72 h IC_50_ (µM)**	**^ *p*-Value**	**^#^ *p*-Value**
100% Fusion-negative	0.298 ± 0.087	-	-	0.357 ± 0.099	-	-	0.288 ± 0.068	-	-
100% Fusion-positive	1.051 ± 0.245	0.022	-	1.296 ± 0.368	0.036	-	1.158 ± 0.211	0.008	-
25:75% Fusion-negative/positive	0.387 ± 0.057	NS	0.027	0.464 ± 0.073	NS	0.048	0.343 ± 0.084	NS	0.012
50:50% Fusion-negative/positive	0.399 ± 0.096	NS	*NS*	0.348 ± 0.075	NS	0.032	0.329 ± 0.033	NS	0.008
75:25% Fusion-negative/positive	0.313 ± 0.119	NS	0.031	0.285 ± 0.078	NS	0.025	0.201 ± 0.050	NS	0.005
**Doxorubicin**	**Clone D (Fus − ve) and Clone 15B (Fus + ve)**			**Clone E (Fus − ve) and Clone 9 (Fus + ve)**			**Clone E (Fus − ve) and Clone 18B (Fus + ve)**		
	**72 h IC_50_ (nM)**	**^ *p*-Value**	**^#^ *p*-Value**	**72 h IC_50_ (nM)**	**^ *p*-Value**	**^#^ *p*-Value**	**72 h IC_50_ (nM)**	**^ *p*-Value**	**^#^ *p*-Value**
100% Fusion-negative	16.174 ± 5.165	-	-	31.373 ± 6.600	-	-	43.532 ± 6.878	-	-
100% Fusion-positive	44.062 ± 4.500	0.007	-	182.293 ± 57.578	0.001	-	112.613 ± 28.684	0.034	-
25:75% Fusion-negative/positive	27.061 ± 3.916	NS	0.029	36.423 ± 7.845	NS	0.046	46.339 ± 6.600	NS	0.040
50:50% Fusion-negative/positive	21.580 ± 2.115	NS	0.004	41.369 ± 6.141	NS	0.002	53.862 ± 10.862	NS	*NS*
75:25% Fusion-negative/positive	16.203 ± 3.854	NS	0.003	23.714 ± 9.745	NS	0.035	42.472 ± 10.916	NS	0.039
**Paclitaxel**	**Clone D (Fus − ve) and Clone 15B (Fus + ve)**			**Clone E (Fus − ve) and Clone 9 (Fus + ve)**			**Clone E (Fus − ve) and Clone 18B (Fus + ve)**		
	**72 h IC_50_ (nM)**	**^ *p*-Value**	**^#^ *p*-Value**	**72 h IC_50_ (nM)**	**^ *p*-Value**	**^#^ *p*-Value**	**72 h IC_50_ (nM)**	**^ *p*-Value**	**^#^ *p*-Value**
100% Fusion-negative	0.769 ± 0.243	-	-	0.663 ± 0.203	-	-	1.001 ± 0.149	-	-
100% Fusion-positive	7.902 ± 0.028	0.028	-	86.189 ± 33.774	0.045		44.035 ± 16.859	0.028	-
25:75% Fusion-negative/positive	2.316 ± 0.537	0.039	NS	1.403 ± 0.407	NS	0.024	2.799 ± 0.525	0.017	0.033
50:50% Fusion-negative/positive	0.995 ± 0.285	NS	0.032	1.026 ± 0.231	NS	0.024	1.297 ± 0.345	NS	0.029
75:25% Fusion-negative/positive	1.096 ± 0.396	NS	0.034	1.025 ± 0.385	NS	0.024	1.157 ± 0.570	NS	0.029

Data represent mean IC_50_ ± SEM from a minimum of 3 independent experiments. Viability determined through DAPI staining 72 h post drug treatment with ABCB1 non-substrate (cisplatin) or substrates (doxorubicin, paclitaxel). ^ *p*-value compared to 100% fusion-negative (Fus – ve) clone. ^#^ *p*-value compared to 100% fusion-positive (Fus + ve) clone. NS: Not significant.

**Table 2:** FDA compounds that induce cytotoxicity regardless of *SLC25A40-ABCB1* fusion status.

Compound ID	Class	Mechanism of Action	Transporter *	GR_50_ (µM)								
				Clone D	Clone E	Clone 9	Clone 18B	Clone D:9 (50:50 Mix)	Fusion Positive Average	Fusion Negative Average	# *p*-Value	^ *p*-Value
4-demethyl-epicro-podophyllotoxin	Lignan	Antimitotic, binds α- and β-tubulin	**ABCB1**	0.156	0.179	0.136	0.100	0.170	0.167	0.118	NS	NS
7-ethyl-10-hydroxycamptothecin	Alkaloid	Inhibition of DNA topoisomerase I	**ABCB1**, ABCG2	0.066	0.076	0.006	0.043	0.053	0.071	0.024	NS	NS
amsacrine	Acridine	DNA intercalation and inhibition of topoisomerase II	**ABCB1**	0.057	0.134	0.027	0.048	0.056	0.095	0.037	NS	NS
bleomycin	Antibiotic	Inhibition of DNA synthesis		0.298	0.219	0.505	0.553	0.353	0.258	0.529	0.028	NS
camptothecin	Alkaloid	Inhibition of DNA topoisomerase I	**ABCB1**, ABCG2	0.108	0.109	0.047	0.070	0.089	0.108	0.059	NS	NS
cedrelone	Limonoid			1.147	1.269	1.833	1.012	0.973	1.208	1.422	NS	NS
cerivastatin lactone	Statin	Competitive HMG-CoA reductase inhibitor	ABCB1, ABCC2, ABCG2, ABC11, SLCO1B1	0.299	0.265	0.160	0.209	0.251	0.282	0.185	NS	NS
colchicine	Alkaloid	Inhibition of inflammation caused by tubulin disruption	**ABCB1**	UTBD	UTBD	UTBD	UTBD	UTBD	-	-	-	-
convallatoxin	Cardiac glycoside	Inhibition of Na^+^/K^+^- ATPase	**ABCB1**	0.017	0.018	0.011	0.029	0.017	0.018	0.020	NS	NS
cytarabine	Antimetabolite	Pyrimidine nucleoside	ABCC10, SLC22A4, SLC29A1	0.036	0.036	0.018	0.067	0.044	0.036	0.043	NS	NS
dactinomycin	Antibiotic	DNA intercalation	**ABCB1**, ABCC6, ABCC1, ABCG2, SLC22A5,	1.207	1.490	0.589	1.086	1.032	1.348	0.837	NS	NS
dasatinib	Tyrosine kinase	BCR/ABL and Src family tyrosine kinase inhibitor	**ABCB1**, ABCG2	0.060	0.117	0.090	0.035	0.081	0.089	0.062	NS	NS
digitoxin	Cardiac glycoside	Inhibition of Na^+^/K^+^- ATPase	SLCO1A2, SLCO4C1	0.403	0.400	0.388	0.373	0.413	0.402	0.380	NS	NS
docetaxel	Taxoid	Antimitotic, binds tubulin beta-1 chain	**ABCB1**, ABCC10, ABCG2, ABCC1, ABCC2, SLCO1B3, SLC22A7,	UTBD	UTBD	UTBD	UTBD	UTBD	-	-	-	-
emetine dihydrochloride	Antiprotozoal agent and emetic	Inhibition of protein synthesis		0.009	0.036	0.036	0.043	0.012	0.023	0.040	NS	NS
gentian_violet	Antifungal	Mitotic poison	SLC22A1	0.170	0.319	0.311	0.350	0.235	0.245	0.331	NS	NS
gramicidin	Antibiotic	Membrane disruption and permeabilization (Gram-positive bacteria)	**ABCB1**	0.382	0.396	0.444	0.487	0.405	0.389	0.465	NS	NS
harringtonine	Cephalotaxine alkaloid	Inhibition of protein synthesis		0.042	0.049	0.026	0.050	0.035	0.046	0.038	NS	NS
hexachlorophene	Chlorinated bisphenol antiseptic	Inhibition of respiratory D-lactate dehydrogenase		3.855	2.758	1.658	1.911	3.330	3.306	1.784	NS	NS
irinotecan_hcl trihydrate	Antineoplastic	Inhibition of DNA topoisomerase I	**ABCB1**, ABCC1, ABCG2, ABCC2, SLC22A3, SLCO1B1	0.234	0.125	0.006	0.024	0.113	0.179	0.015	NS	NS
irinotecan_hydrochloride	Antineoplastic	Inhibition of DNA topoisomerase I	**ABCB1**, ABCC1, ABCG2, ABCC2, SLC22A3, SLCO1B1	0.604	0.227	0.012	0.060	0.246	0.415	0.036	NS	NS
lanatoside c	Cardiac glycoside			0.133	0.124	0.116	0.125	0.129	0.128	0.121	NS	NS
mitoxanthrone hcl	Anthracenediones	DNA Intercalation	**ABCB1**, ABCC1, ABCG2	0.000	0.001	0.001	0.000	0.000	0.000	0.000	NS	NS
nocodazole	Antineoplastic	Inhibition of microtubule polymerization		0.087	0.111	0.100	0.079	0.081	0.099	0.090	NS	NS
ouabain_octahydrate	Cardioactive glycoside	Inhibition of Na^+^/K^+^- ATPase	SLCO1A2, SLC22A8, SLCO4C1, SLCO1B3, SLCO1C1, SLCO1B1	0.058	0.043	0.060	0.062	0.053	0.050	0.061	NS	NS
parthenolide	Sesquiterpene lactone	Inhibition of IkB kinase (IKK) and IKKβ		0.332	0.583	0.220	0.343	0.228	0.457	0.281	NS	NS
patulin	Polyketide mycotoxin			0.147	0.236	0.095	0.219	0.091	0.191	0.157	NS	NS
podofilox	Lignan	Inhibition of DNA topoisomerase II		UTBD	UTBD	UTBD	UTBD	UTBD	-	-	-	-
podophyllin_acetate	Keratolytic	Binds to tubulin to prevent formation of microtubules		0.065	0.079	0.067	0.053	0.066	0.072	0.060	NS	NS
proscillaridin a	Cardioactive glycoside	Inhibition of Na^+^/K^+^- ATPase		0.008	0.008	0.006	0.011	0.012	0.008	0.009	NS	NS
pyrithione_zinc	Antimicrobial	Copper-mediated loss of function of iron–sulphur proteins		0.440	0.423	0.731	1.056	0.382	0.432	0.893	NS	NS
strophanthidin acetate	Cardiac glycoside	Inhibition of Na^+^/K^+^- ATPase		0.349	0.202	0.250	0.302	0.232	0.275	0.276	NS	NS
teniposide	Antineoplastic	Inhibition of DNA topoisomerase II	ABCC6, ABCG2	0.039	0.065	0.005	0.032	0.018	0.052	0.019	NS	NS
topotecan_hydrochloride	Antineoplastic	Inhibition of DNA topoisomerase I	**ABCB1**, ABCG2, SLC47A1, SLC47A2,	0.093	0.105	0.023	0.073	0.084	0.099	0.048	NS	NS
triptolide	Diterpenoid epoxide	Inhibits transcription and nucleotide excision repair activity of RNA polymerase II		0.012	0.007	0.006	0.006	0.008	0.010	0.006	NS	NS
vinblastine sulfate	Vinca alkaloid	Inhibition of microtubule polymerization	ABCB1, ABCC1, ABCC2, ABCC6, ABCB11, SLCO1B1,	0.011	0.021	0.013	0.011	0.012	0.016	0.012	NS	NS

# GR_50_ comparison of *SLC25A40-ABCB1* fusion-negative (Clone D, Clone E) versus fusion-positive clones (Clone 9, Clone 18B). ^ GR_50_ comparison of *SLC25A40-ABCB1* fusion-negative (Clone D, Clone E) clones and mixed-fusion Clone D:Clone 9 (50:50) population. UTBD: Unable to be determined (flat GR dose curve). NS: Not significant. * Transporter data sourced from DrugBank Online (https://go.drugbank.com, accessed on 24 June 2021).

## Data Availability

AOCS18.5 fusion-negative and fusion-positive cell line RNAseq data are available in the GEO database (http://www.ncbi.nlm.nih.gov/gdsunder, accessed on 3 September 2021, accession number GSE183210. The Patch et al. data is available from the European Genome-phenome Archive (EGA) repository under the accession code EGAD00001000877.

## Supplementary Materials

The following are available online at https://www.mdpi.com/article/10.3390/cancers13225644/s1, Figure S1: *SLC25A40-ABCB1* and *ABCB1* expression levels in AOCS18.5 single-cell clones, Figure S2: *SLC25A40-ABCB1* fusion-positive cells demonstrate increased efflux of *ABCB1* substrates, Figure S3: *SLC25A40-ABCB1* fusions drive resistance to standard of care HGSOC chemotherapeutics, Figure S4: Mutational analysis of AOCS18.5 lines identifies similarities and differences between fusion-positive and negative clones, Figure S5: *SLC25A40-ABCB1* fusion-driven transcriptional profiles in HGSOC patient samples, Figure S6: Fusion-negative cells outcompete the growth of *ABCB1* fusion-positive clones when co-cultured, Figure S7: Clonal composition influences drug sensitivity, Figure S8: Identification of FDA agents that induce cytotoxicity independent of *SLC25A40-ABCB1* fusion status, Figure S9: FDA agents primarily induce G0-G1 and above G2-M arrest in AOCS18.5 clones, Table S1: Proliferation rates of AOCS18.5 fusion-negative and positive clones, Table S2: Drug hits from primary FDA screening
