# Supplementary material for: Phenotypic Consequences of SLC25A40-ABCB1 Fusions beyond Drug Resistance in High-Grade Serous Ovarian Cancer
Source: Cancers (Basel). 2021 Nov 11;13(22):5644. doi: 10.3390/cancers13225644 (PMC8616176; doi:10.3390/cancers13225644)
Supplement: Supplementary file 1 [file cancers-13-05644-s001.zip › Supplementary Figure S1 mRNA and protein levels_Version 2_11.11.2021.pdf]

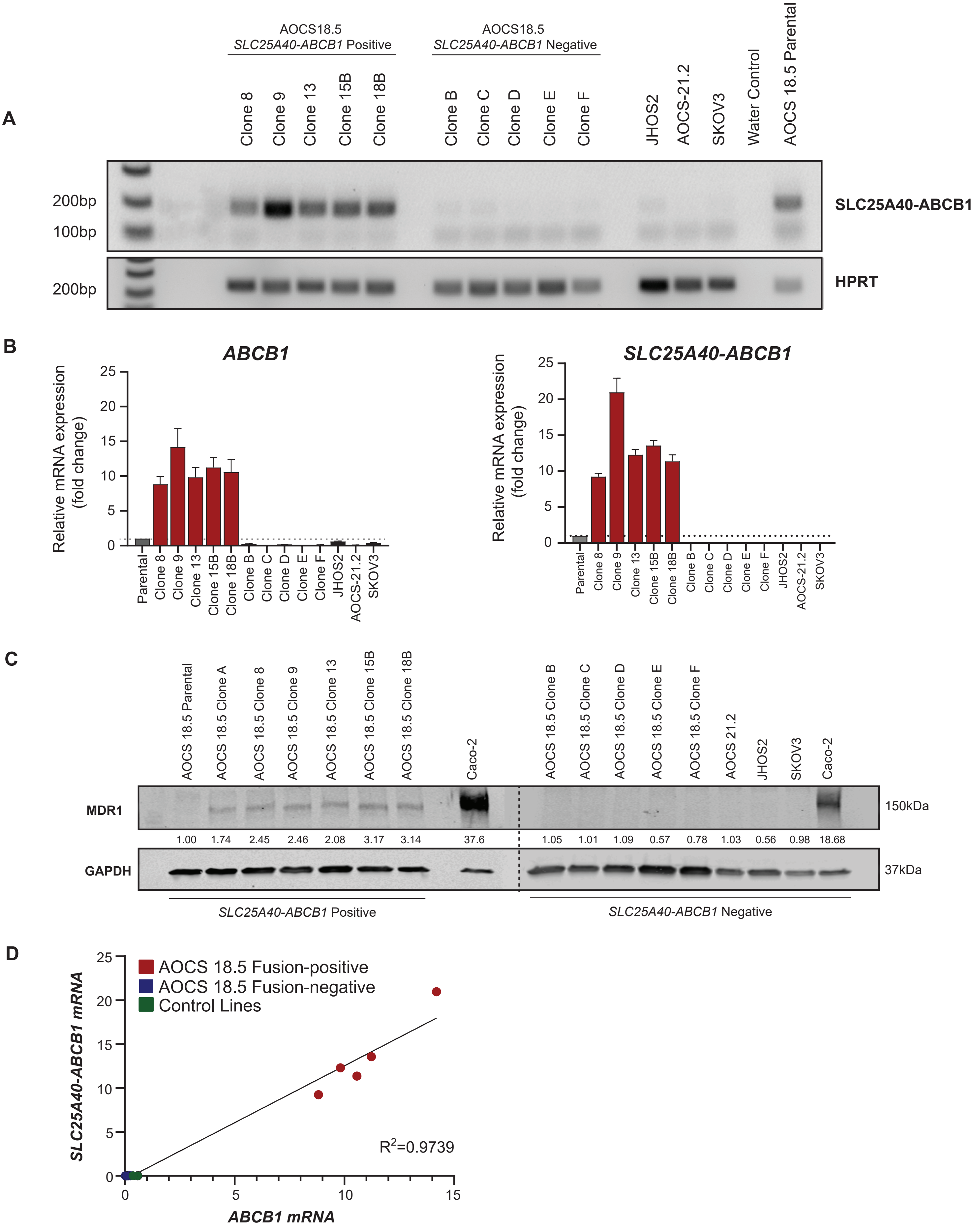

Supplementary Fig. S1: SLC25A40-ABCB1 and ABCB1 expression levels in AOCS18.5 single cell clones.

(A) DNA detection (PCR) of the SLC25A40-ABCB1 breakpoint in AOCS18.5 derived single cell clones and fusion-negative HGSOC control cell lines JHOS2, AOCS-21.2 and SKOV3. Expected amplicon size, 154bp. HPRT used as a loading control. (B) Relative mRNA expression of *SLC25A40-ABCB1* and total *ABCB1* determined through qRT-PCR. AOCS18.5 fusion-positive single cell clones denoted in red, with expression normalized to AOCS18.5 parental cells. Data represent mean expression ± SEM from three independent experiments. (C) Western blot analysis of MDR1 (ABCB1) expression. Caco-2, known *SLC25A40-ABCB1* fusion-negative, ABCB1 overexpressing colon cell line used for MDR1 reference. GAPDH used as loading control. ABCB1 densitometry normalized to GAPDH and relative to AOCS18.5 parental cells is depicted. (D) Correlation between basal AB*CB1* and *SLC25A40-ABCB1* mRNA expression in AOCS18.5 cell lines utilised in this study.
