# Supplementary material for: Phenotypic Consequences of SLC25A40-ABCB1 Fusions beyond Drug Resistance in High-Grade Serous Ovarian Cancer
Source: Cancers (Basel). 2021 Nov 11;13(22):5644. doi: 10.3390/cancers13225644 (PMC8616176; doi:10.3390/cancers13225644)
Supplement: Supplementary file 1 [file cancers-13-05644-s001.zip › Supplementary Figure S2 MDR1 Assay.pdf]

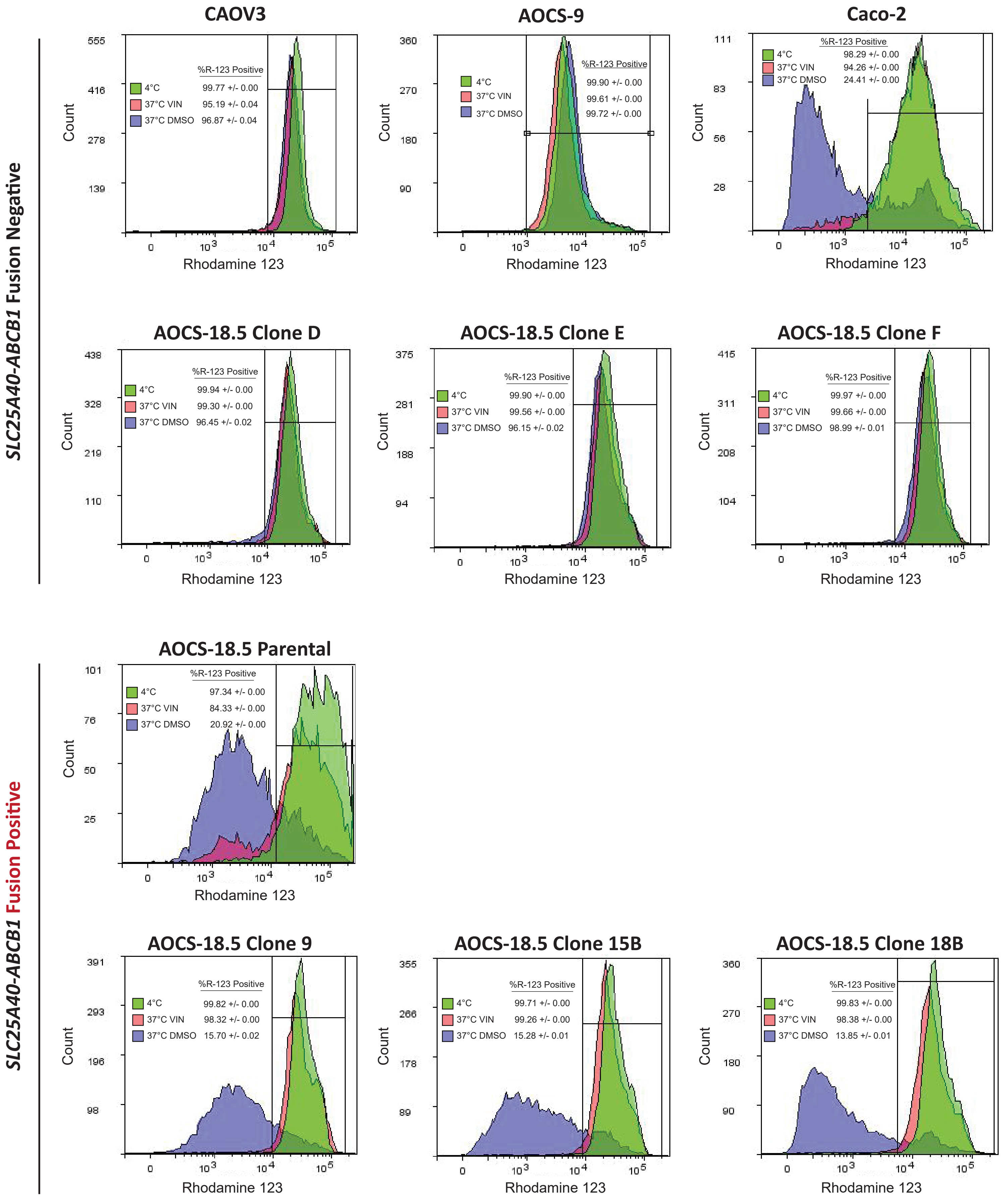

Supplementary Fig. S2: *SLC25A40-ABCB1* fusion-positive cells demonstrate increased efflux of ABCB1 substrates

Representative flow cytometric analysis of rhodamine 123 efflux via P-gp in AOCS18.5 parental, and *SLC25A40-ABCB1* fusion-negative (Clone D, E, F) and fusion-positive (Clone 9, 15B, 18B) clones. CAOV3 and AOCS-9 used as fusion-negative HGSOC control lines. Caco-2, known *SLC25A40-ABCB1* fusion-negative, *ABCB1* overexpressing colon intestinal cell line used for *ABCB1* overexpression comparison. Cells were incubated with rhodamine 123 for 1hr (4°C) followed by the following treatments: 4°C (green, P-gp inactive), 37°C with ABCB1 competitive substrate Vinblastine (VIN) (pink), and 37°C DMSO (pink, P-gp active). Efflux was quantified in terms of the fraction of cells in the M1 region of the plot (low fluorescence). Data represents mean rhodamine 123 cellular retention ± STDEV from two independent experiments.
