# Supplementary material for: Phenotypic Consequences of SLC25A40-ABCB1 Fusions beyond Drug Resistance in High-Grade Serous Ovarian Cancer
Source: Cancers (Basel). 2021 Nov 11;13(22):5644. doi: 10.3390/cancers13225644 (PMC8616176; doi:10.3390/cancers13225644)
Supplement: Supplementary file 1 [file cancers-13-05644-s001.zip › Supplementary Figure S3.pdf]

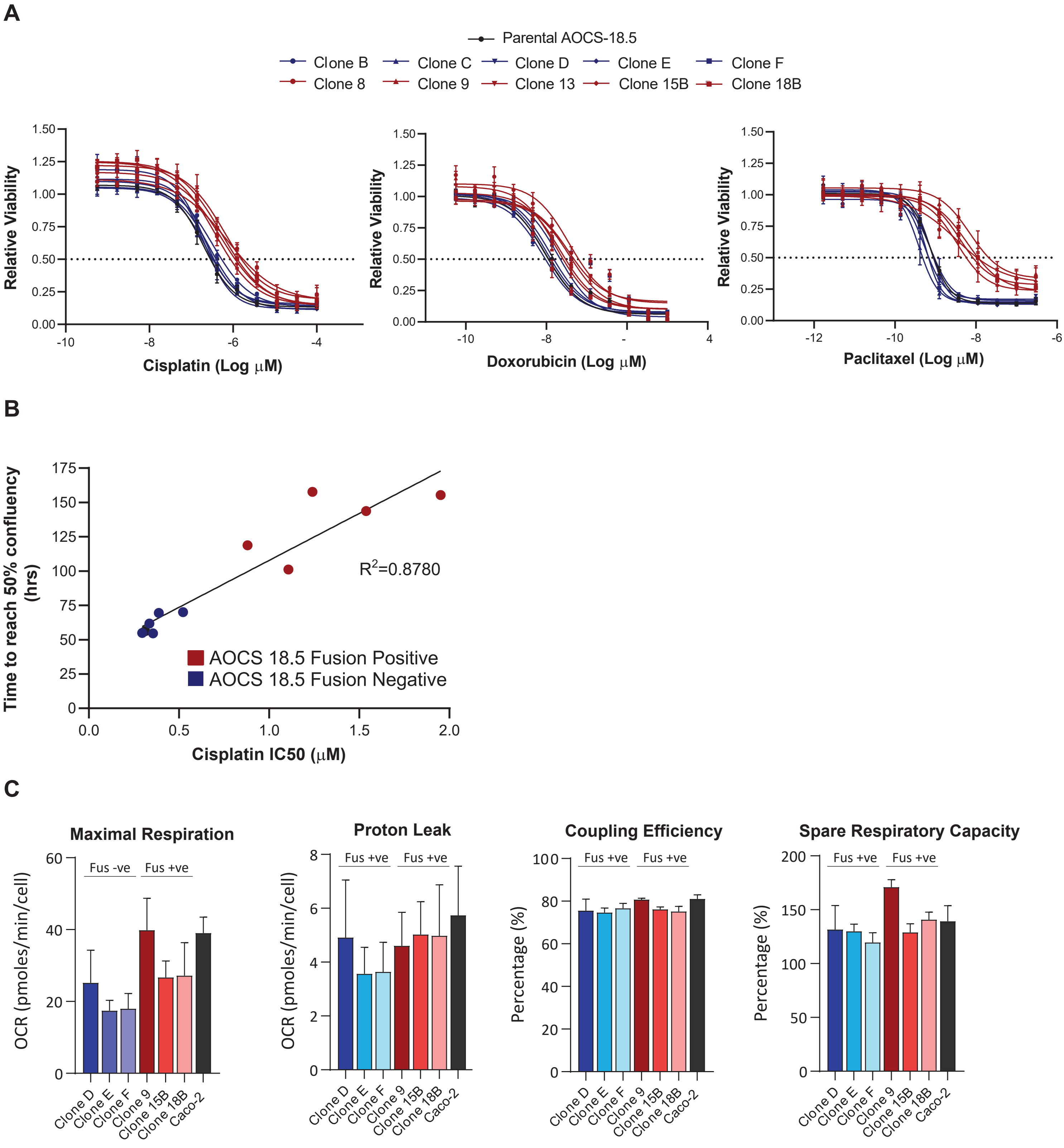

Supplementary Fig. S3: *SLC25A40-ABCB1* fusions drive resistance to standard of care HGSOC chemotherapeutics

(A) Relative viability of AOCS18.5 parental (black), fusion-negative (blue) and fusion-positive (red) clones following 72hr treatment with ABCB1 substrate (Doxorubicin, Paclitaxel) and non-substrate (Cisplatin) therapy. Viability assessed through DAPI staining. Data represents mean viability from DMSO control ± SEM from a minimum of three independent experiments. Dashed line denotes IC_{50}. (B) Correlation between AOCS18.5 fusion-negative (blue, n=5), fusion-positive (red, n=5) clone Cisplatin IC_{50} and proliferation rate (time to reach 50% confluence, IncuCyte assay). (C) Maximal respiration, proton leak, coupling efficiency and spare respiratory capacity of fusion-negative and positive clones evaluated through oxygen consumption rate (OCR) analysis (Seahorse extracellular flux assay). The mitochondrial stress test was used to obtain bioenergetics parameters, by adding the ATP synthase inhibitor Oligomycin A (1 µM), to derive ATP-linked OCR, FCCP (1µM) to uncouple the mitochondria for maximal OCR, and Antimycin A/ Rotenone (1 µM), to shut down electron transport chain function. Data normalised to cell number (Hoechst staining). Caco-2, known *ABCB1* overexpressing colon intestinal cell line (*SLC25A40-ABCB1* fusion negative). Data represents mean ± SEM from a minimum of three independent experiments.
