# Supplementary material for: Phenotypic Consequences of SLC25A40-ABCB1 Fusions beyond Drug Resistance in High-Grade Serous Ovarian Cancer
Source: Cancers (Basel). 2021 Nov 11;13(22):5644. doi: 10.3390/cancers13225644 (PMC8616176; doi:10.3390/cancers13225644)
Supplement: Supplementary file 1 [file cancers-13-05644-s001.zip › Supplementary Figure S4 Targeted sequencing.pdf]

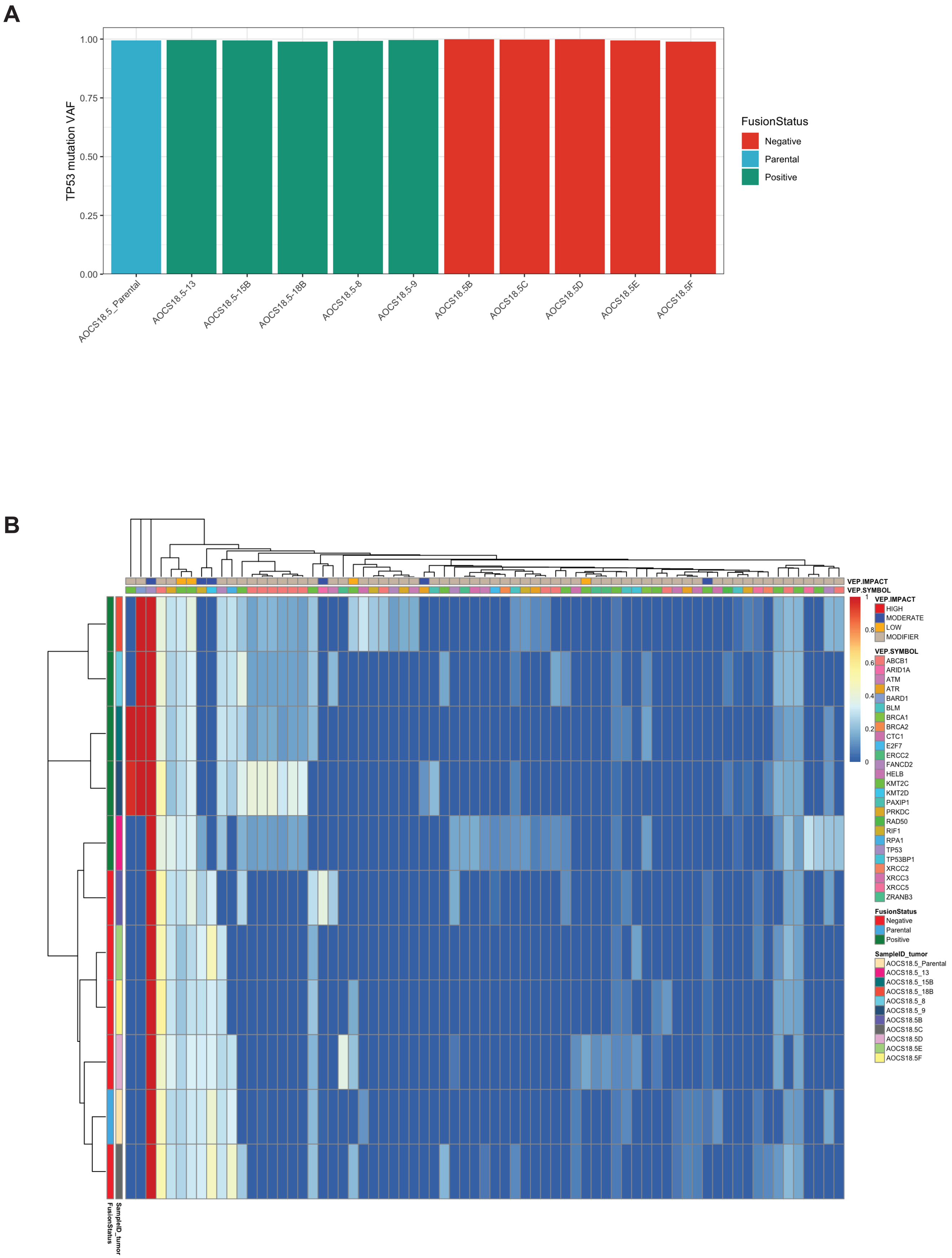

Supplementary Fig. S4. Mutational analysis of AOCS18.5 lines identifies similarities and differences between fusion positive and negative clones.

(A) A single high variant (ENSP00000269305.4:p.Lys132Met) was identified in TP53 in all AOCS18.5 lines (parental and clones), with variant allele frequencies (VAF) >98%. (B) A heatmap of the VAF of all mutations detected sees the fusion positive clones clustering together and the fusion negative clones clustering with the parental line.
