# Supplementary material for: Phenotypic Consequences of SLC25A40-ABCB1 Fusions beyond Drug Resistance in High-Grade Serous Ovarian Cancer
Source: Cancers (Basel). 2021 Nov 11;13(22):5644. doi: 10.3390/cancers13225644 (PMC8616176; doi:10.3390/cancers13225644)
Supplement: Supplementary file 1 [file cancers-13-05644-s001.zip › Supplementary Figure S5 RNA seq.pdf]

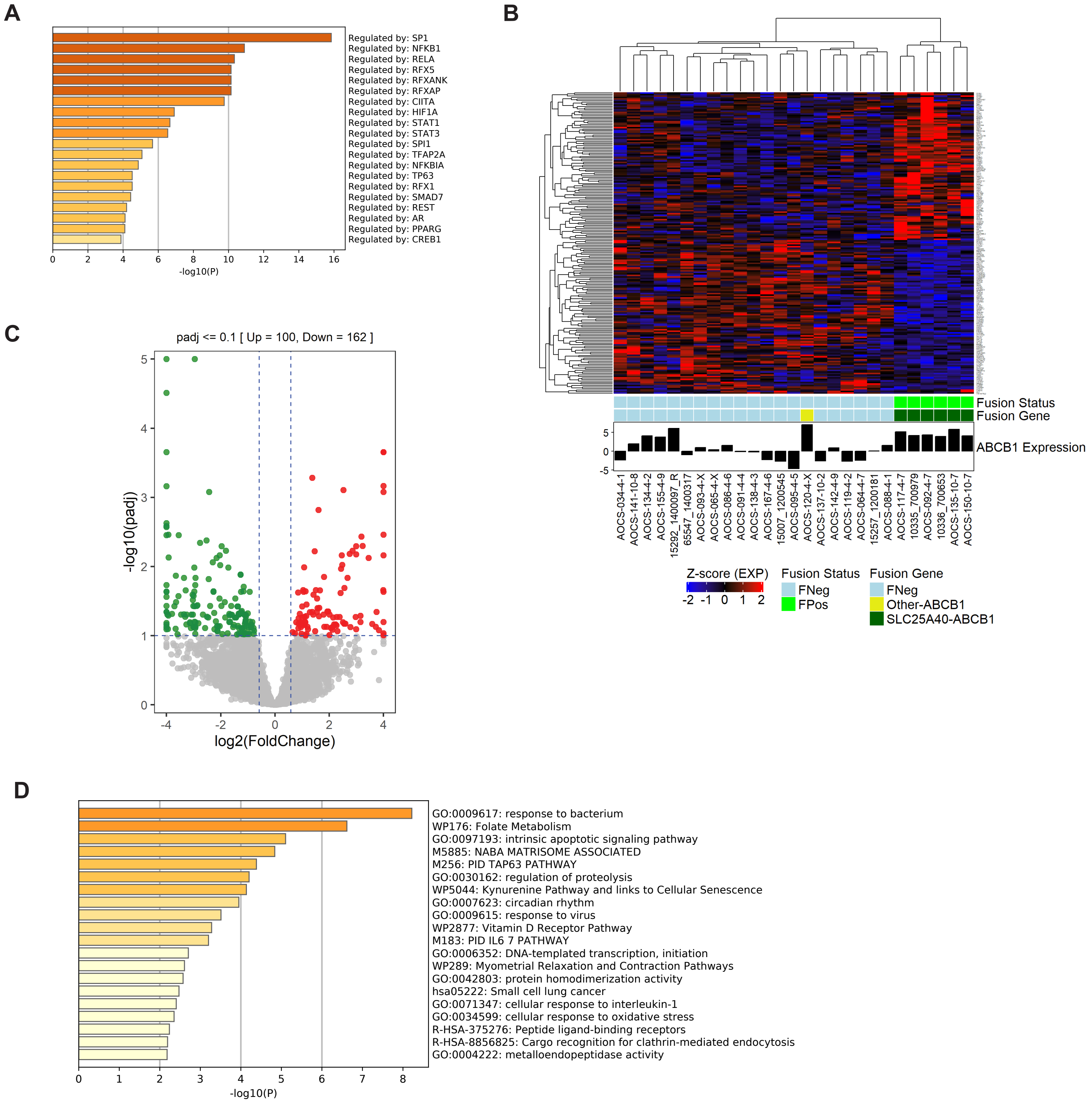

Supplementary Fig. S5: *SLC25A40-ABCB1* fusion driven transcriptional profiles in HGSOC patient samples

(A) Metascape TRUSST analysis of transcriptional regulatory relationships significantly enriched in AOCS18.5 fusion-positive clones. (B) Unsupervised hierarchical clustering analysis (basal transcriptional profile) of *SLC25A40-ABCB1* fusion-negative (n=21) and fusion-positive (n=6) relapsed HGSOC patient ascites samples. The top 100 significantly differentially expressed genes from RNA-seq analysis are shown as well as *ABCB1* expression. Sample AOCS_120_4_X contains an alternative transcriptional start site in exon 2 of ABCB1 due to a large insertion in intron 1 of ABCB1. (C) Volcano plot showing differentially expressed genes significantly up/down regulated in fusion-positive clones compared to fusion-negative. (D) Metascape pathway analysis of significantly induced genes upregulated by *SLC25A40-ABCB1* fusions in HGSOC patient samples. Cutoff criteria for all analysis was Log2 fold change of 1.5 and p-adj of <0.1.
