# Supplementary material for: Phenotypic Consequences of SLC25A40-ABCB1 Fusions beyond Drug Resistance in High-Grade Serous Ovarian Cancer
Source: Cancers (Basel). 2021 Nov 11;13(22):5644. doi: 10.3390/cancers13225644 (PMC8616176; doi:10.3390/cancers13225644)
Supplement: Supplementary file 1 [file cancers-13-05644-s001.zip › Supplementary Figure S6 v2 Coculture Experiments.pdf]

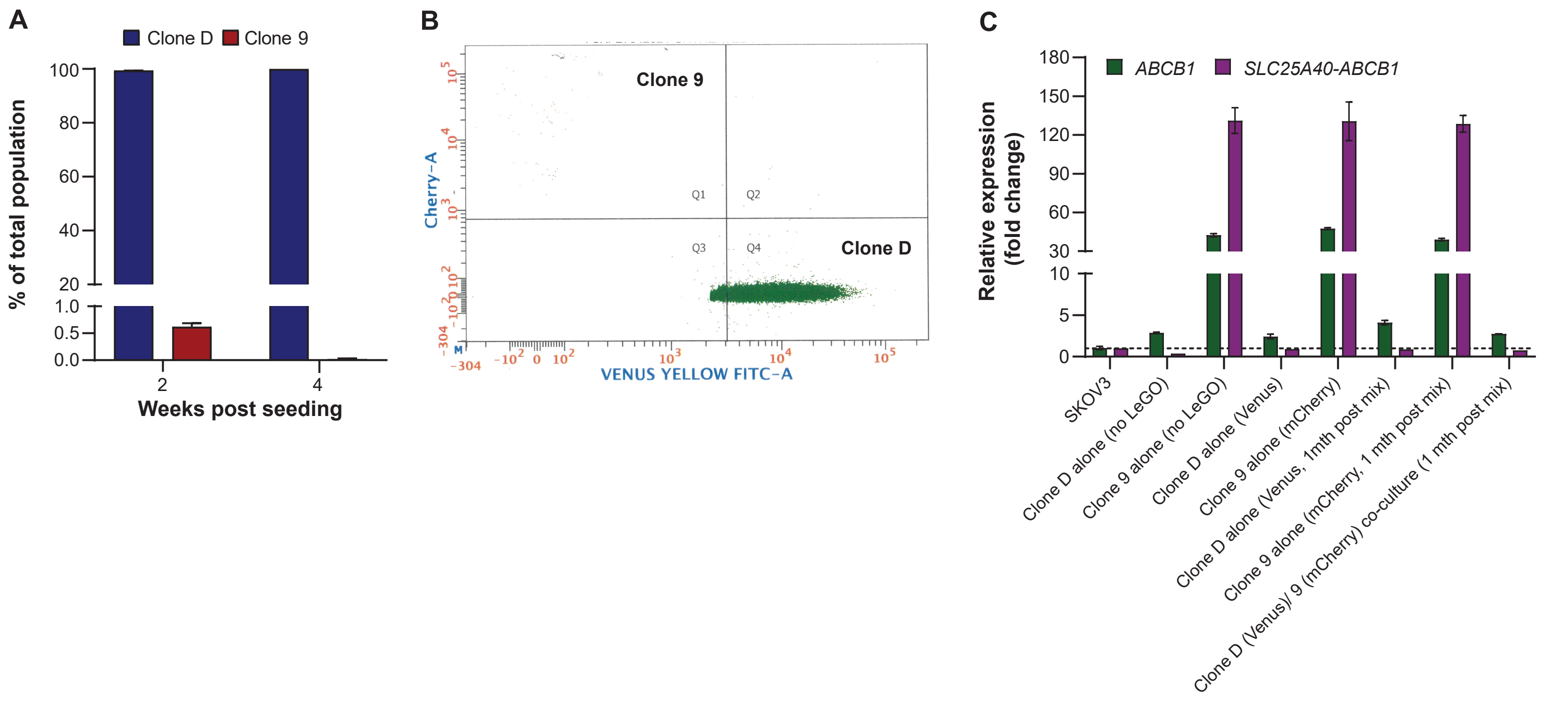

Supplementary Fig. S6: Fusion-negative cells outcompete the growth of *ABCB1* fusion-positive clones when co-cultured

(A) Percentage of total population of AOCS18.5 Clone D (fusion-negative, venus yellow) and Clone 9 (fusion-positive, mCherry) cells following two and four weeks post co-culture (50:50 mix). Data represents mean percentage ± STDEV from duplicate flasks ascertained from FACS analysis. (B) Representative flow cytometry plot of venus yellow (Q4) and mCherry (Q1) cell sorting, two weeks post co-culture of Clone D and Clone 9 cells. (C) qRT-PCR analysis of *ABCB1* (green) and *SLC25A40-ABCB1* (purple) expression normalized to fusion-negative HGSOC SKOV3 cells. Data represents mean expression ± STDEV from duplicate flasks. Transduction of Clone D and Clone 9 cells with LeGO vectors had no effect on basal *ABCB1* and *SLC25A40-ABCB1* expression levels.
