# Supplementary material for: Phenotypic Consequences of SLC25A40-ABCB1 Fusions beyond Drug Resistance in High-Grade Serous Ovarian Cancer
Source: Cancers (Basel). 2021 Nov 11;13(22):5644. doi: 10.3390/cancers13225644 (PMC8616176; doi:10.3390/cancers13225644)
Supplement: Supplementary file 1 [file cancers-13-05644-s001.zip › Supplementary Figure S7 Clonal mixing and IC50.pdf]

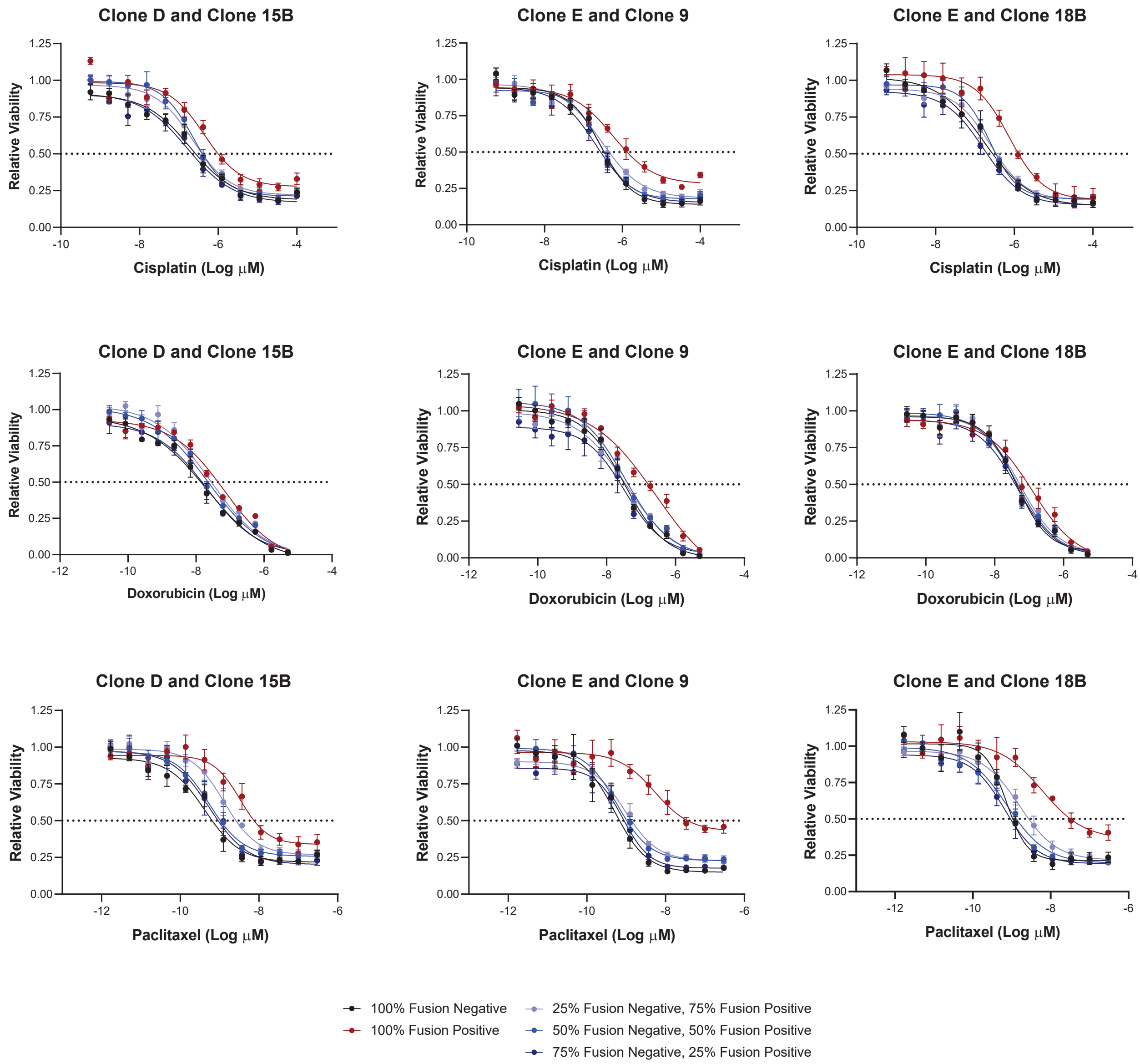

Supplementary Fig. S7: Clonal composition influences drug sensitivity

SLC25A40-ABCB1 fusion negative (Clone D, Clone E) and fusion-positive (Clone 9, Clone 18B) clones were seeded alone or co-cultured in three different ratios (25:75, 50:50, 75:25) and treated with Cisplatin, Doxorubicin or Paclitaxel for 72hrs. Relative viability normalized to DMSO control was determined through DAPI staining. Data represents relative viability ± SEM from a minimum of four independent experiments. Line denotes IC_{50}.
