# Supplementary material for: Phenotypic Consequences of SLC25A40-ABCB1 Fusions beyond Drug Resistance in High-Grade Serous Ovarian Cancer
Source: Cancers (Basel). 2021 Nov 11;13(22):5644. doi: 10.3390/cancers13225644 (PMC8616176; doi:10.3390/cancers13225644)
Supplement: Supplementary file 1 [file cancers-13-05644-s001.zip › Supplementary Figure S8 GR profiles FDA.pdf]

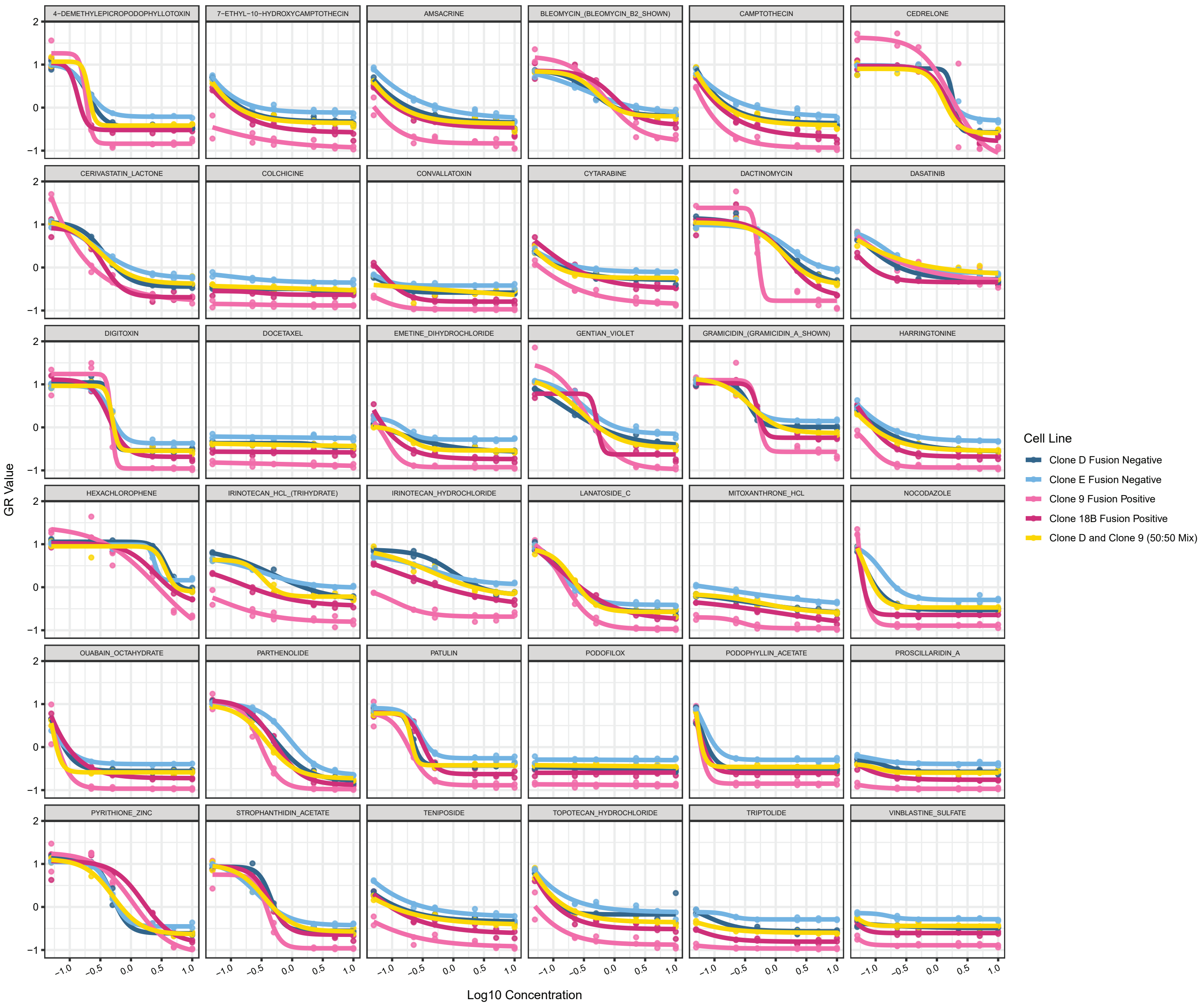

Supplementary Fig. S8: Identification of FDA agents that induce cytotoxicity independent of *SLC25A40-ABCB1* fusion status.

Growth rate inhibition (GR) metrics normalized to DMSO control of AOCS18.5 fusion-negative (Clone D, Clone E), fusion-positive (Clone 9 and Clone 18B) and 50:50 mixed cellular populations (Clone D and Clone 9) treated with 36 FDA approved agents. GR determined through DAPI staining 72hrs post treatment (10, 5, 2.25, 0.5, 0.225, 0.05 µM) from duplicate wells.
