# Supplementary material for: Phenotypic Consequences of SLC25A40-ABCB1 Fusions beyond Drug Resistance in High-Grade Serous Ovarian Cancer
Source: Cancers (Basel). 2021 Nov 11;13(22):5644. doi: 10.3390/cancers13225644 (PMC8616176; doi:10.3390/cancers13225644)
Supplement: Supplementary file 1 [file cancers-13-05644-s001.zip › Supplementary Table S1 Incucyte Proliferation.pdf]

Supplementary Table S1: Proliferation rates of AOCS-18.5 fusion-negative and positive clones

AOCS-18.5 Line	Fusion Status	Time to reach 50% confluence (hrs)
Parental	Positive[#]	56.15 ± 3.75
Clone B	Negative	70.06 ± 5.73
Clone C	Negative	69.42 ± 2.11
Clone D	Negative	61.81 ± 3.32
Clone E	Negative	54.97 ± 4.67
Clone F	Negative	54.66 ± 4.09
Clone 8	Positive	155.38 ± 24.94
Clone 9	Positive	143.66 ± 10.14
Clone 13	Positive	118.79 ± 6.41
Clone 15B	Positive	101.18 ± 2.88
Clone 18B	Positive	157.70 ± 10.95

Cell line comprises approximately 40% fusion positive cells.
Data represents mean confluence ± SEM from a minimum of five independent IncuCyte experiments
