# Supplementary material for: Phenotypic Consequences of SLC25A40-ABCB1 Fusions beyond Drug Resistance in High-Grade Serous Ovarian Cancer
Source: Cancers (Basel). 2021 Nov 11;13(22):5644. doi: 10.3390/cancers13225644 (PMC8616176; doi:10.3390/cancers13225644)
Supplement: Supplementary file 1 [file cancers-13-05644-s001.zip › Supplementary Table S2.pdf]

**Supplementary Table 2:** Drug hits from primary FDA screening.

| Hit | QCL Sample Number | Compound ID | # Hit Dose Points |
|---|---|---|---|
| **1** | **SN01006065** | **TRIPTOLIDE** | **3** |
| **2** | **SN01004975** | **TENIPOSIDE** | **3** |
| **3** | **SN01005284** | **PROSCILLARIDIN_A** | **3** |
| **4** | **SN01005428** | **PODOFILOX** | **3** |
| **5** | **SN00852776** | **PACLITAXEL** | **3** |
| **6** | **SN00846426** | **HARRINGTONINE** | **3** |
| **7** | **SN01004313** | **EMETINE_DIHYDROCHLORIDE** | **3** |
| **8** | **SN00852833** | **DOCETAXEL** | **3** |
| **9** | **SN01005867** | **CONVALLATOXIN** | **3** |
| **10** | **SN01004261** | **COLCHICINE** | **3** |
| **11** | **SN00852795** | **7-ETHYL-10-HYDROXYCAMPTOTHECIN** | **3** |
| **12** | **SN00852856** | **10-HYDROXYCAMPTOTHECIN** | **3** |
| 13 | SN00844674 | VINCRISTINE_SULFATE | 2 |
| 14 | SN01006021 | VERATRINE_HYDROCHLORIDE | 2 |
| 15 | SN01004936 | TOPOTECAN_HYDROCHLORIDE | 2 |
| 16 | SN00844679 | TOPOTECAN_HCL | 2 |
| 17 | SN01006261 | PODOPHYLLIN_ACETATE | 2 |
| 18 | SN01006517 | PATULIN | 2 |
| 19 | SN01005684 | OUABAIN_OCTAHYDRATE | 2 |
| 20 | SN01005436 | NOCODAZOLE | 2 |
| 21 | SN01004872 | MITOXANTRONE_HYDROCHLORIDE | 2 |
| 22 | SN00852733 | MITOXANTHRONE_HCL | 2 |
| 23 | SN01004680 | LANATOSIDE_C | 2 |
| 24 | SN00838677 | IRINOTECAN_HCL_(TRIHYDRATE) | 2 |
| 25 | SN00852772 | DOXORUBICIN_HCL | 2 |
| 26 | SN01004295 | DIGOXIN | 2 |
| 27 | SN01004294 | DIGITOXIN | 2 |
| 28 | SN00852835 | DASATINIB | 2 |
| 29 | SN01005375 | DACTINOMYCIN | 2 |
| 30 | SN01004272 | CYTARABINE | 2 |
| 31 | SN00838624 | CERIVASTATIN_NA | 2 |
| 32 | SN00838664 | CERIVASTATIN_LACTONE | 2 |
| 33 | SN01005979 | CAMPTOTHECIN | 2 |
| 34 | SN00838731 | ATROSCINE_HBR | 2 |
| 35 | SN01004861 | AMSACRINE | 2 |
| 36 | SN01005834 | 4-DEMETHYLEPICROPODOPHYLLOTOXIN | 2 |
| 37 | SN00852845 | VORINOSTAT | 1 |
| 38 | SN00844643 | VINORELBINE_BITATRATE | 1 |
| 39 | SN00844658 | VINBLASTINE_SULFATE | 1 |
| 40 | SN01005668 | TIOGUANINE | 1 |
| 41 | SN01004535 | THIMEROSAL | 1 |
| 42 | SN01006263 | TANSHINONE_IIA | 1 |
| 43 | SN01004875 | SULFANILATE_ZINC | 1 |

| | | | |
|---|---|---|---|
| 44 | SN01005894 | STROPHANTHIDINIC_ACID_LACTONE_ACETATE | 1 |
| 45 | SN01006189 | STROPHANTHIDIN_ACETATE | 1 |
| 46 | SN01006499 | STROPHANTHIDIN | 1 |
| 47 | SN01005689 | SECURININE | 1 |
| 48 | SN01006250 | SAPPANONE_A_DIMETHYL_ETHER | 1 |
| 49 | SN01004179 | SANGUINARIUM_CHLORIDE | 1 |
| 50 | SN00846304 | RUBITECAN | 1 |
| 51 | SN01006342 | ROTENONE | 1 |
| 52 | SN01004994 | RIVAROXABAN | 1 |
| 53 | SN01004304 | PYRITHIONE_ZINC | 1 |
| 54 | SN01004633 | PUROMYCIN_DIHYDROCHLORIDE | 1 |
| 55 | SN01004478 | PRIMAQUINE_PHOSPHATE | 1 |
| 56 | SN01004946 | PIROCTONE_OLAMINE | 1 |
| 57 | SN01006529 | PICROPODOPHYLLIN | 1 |
| 58 | SN01004576 | PHENYLMERCURIC_ACETATE | 1 |
| 59 | SN01005833 | PARTHENOLIDE | 1 |
| 60 | SN00846327 | OXYMETHOLONE | 1 |
| 61 | SN01004881 | OXIBENDAZOLE | 1 |
| 62 | SN01004866 | NICLOSAMIDE | 1 |
| 63 | SN01004701 | NARASIN | 1 |
| 64 | SN01004586 | MYCOPHENOLIC_ACID | 1 |
| 65 | SN01004180 | MITOMYCIN | 1 |
| 66 | SN01005160 | METHYLENE_BLUE | 1 |
| 67 | SN01006275 | METHYL_GAMBOGATE | 1 |
| 68 | SN01005253 | MELENGESTROL_ACETATE | 1 |
| 69 | SN00852728 | MEBENDAZOLE | 1 |
| 70 | SN01004855 | LASALOCID_SODIUM | 1 |
| 71 | SN01005276 | IRINOTECAN_HYDROCHLORIDE | 1 |
| 72 | SN01004348 | GRAMICIDIN_(GRAMICIDIN_A_SHOWN) | 1 |
| 73 | SN01005838 | GITOXIGENIN_DIACETATE | 1 |
| 74 | SN01004346 | GENTIAN_VIOLET | 1 |
| 75 | SN01005060 | FLUVASTATIN_SODIUM | 1 |
| 76 | SN01005050 | FLUBENDAZOLE | 1 |
| 77 | SN01004812 | FLOXURIDINE | 1 |
| 78 | SN01004629 | FENBENDAZOLE | 1 |
| 79 | SN00838640 | ETOPOSIDE | 1 |
| 80 | SN01006195 | ERGOSTEROL | 1 |
| 81 | SN00844672 | EPIRUBICIN_HCL | 1 |
| 82 | SN01006396 | DIHYDROCELASTRYL_DIACETATE | 1 |
| 83 | SN01006235 | DEOXYSAPPANONE_B_7,4-DIMETHYL_ETHER | 1 |
| 84 | SN01004276 | DAUNORUBICIN_HYDROCHLORIDE | 1 |
| 85 | SN00852681 | DAUNORUBICIN_HCL | 1 |
| 86 | SN01004714 | CYCLOHEXIMIDE | 1 |
| 87 | SN00838670 | CLOFARABINE | 1 |

| | | | |
|---|---|---|---|
| 88 | SN00846345 | CICLOPIROX_OLAMINE | 1 |
| 89 | SN01006524 | CHLOROTHALONIL | 1 |
| 90 | SN01005579 | CETRIMONIUM_BROMIDE | 1 |
| 91 | SN01006361 | CELASTROL | 1 |
| 92 | SN01006089 | CEDRELONE | 1 |
| 93 | SN01005538 | BRONOPOL | 1 |
| 94 | SN01004904 | BLEOMYCIN_(BLEOMYCIN_B2_SHOWN) | 1 |
| 95 | SN01004713 | AZACITIDINE | 1 |
| 96 | SN01005282 | ATRACURIUM_BESYLATE | 1 |
| 97 | SN01005292 | ARSENIC_TRIOXIDE_DIETHANOLAMINE_SALT | 1 |
| 98 | SN01005978 | ANTIMYCIN_A_(A1_SHOWN) | 1 |
| 99 | SN01005941 | ANTHOTHECOL | 1 |
| 100 | SN01004953 | ALBENDAZOLE | 1 |
| 101 | SN00852805 | ADRIAMYCIN_HCL | 1 |
| 102 | SN01005432 | ACRIFLAVINIUM_HYDROCHLORIDE | 1 |
| 103 | SN01006251 | 7-DESACETOXY-6,7-DEHYDROGEDUNIN | 1 |

Primary screening identified 103 compounds in which normalized cell counts were <50% from control (DMSO) at a minimum of one dose point (0.05, 0.5, 5 μM) in all four cell lines (AOCS18.5 Clone D, E, 9 and 18B). Viability determined through DAPI staining following 72 h of treatment.
