# Supplementary figures and images for: Phenotypic Consequences of SLC25A40-ABCB1 Fusions beyond Drug Resistance in High-Grade Serous Ovarian Cancer
Source: Cancers (Basel). 2021 Nov 11;13(22):5644. doi: 10.3390/cancers13225644 (PMC8616176; doi:10.3390/cancers13225644)

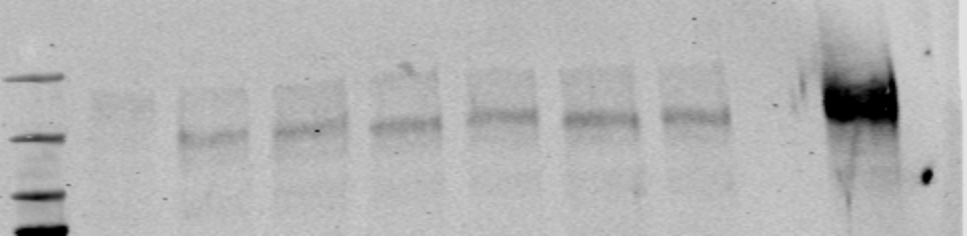

Supplement: Supplementary file 1 [file cancers-13-05644-s001.zip › Original Images of WB in Figure S1/Supplementary Figure 1C Fusion Positive MDR1.tif]

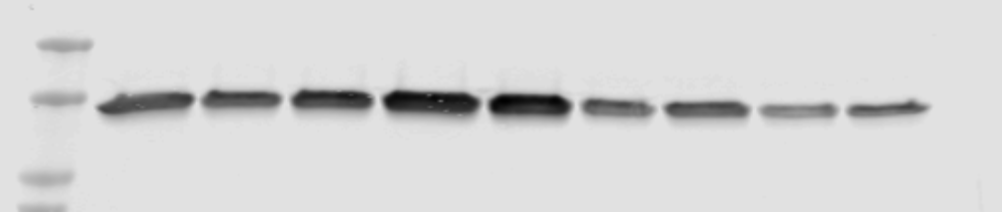

Supplement: Supplementary file 1 [file cancers-13-05644-s001.zip › Original Images of WB in Figure S1/Supplementary Figure 1C GAPDH Fusion Negative.tif]

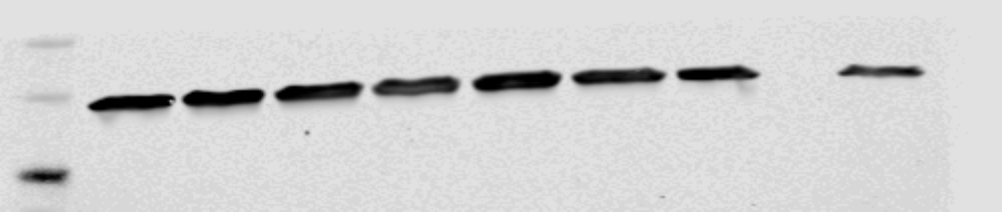

Supplement: Supplementary file 1 [file cancers-13-05644-s001.zip › Original Images of WB in Figure S1/Supplementary Figure 1C GAPDH Fusion Positive.tif]

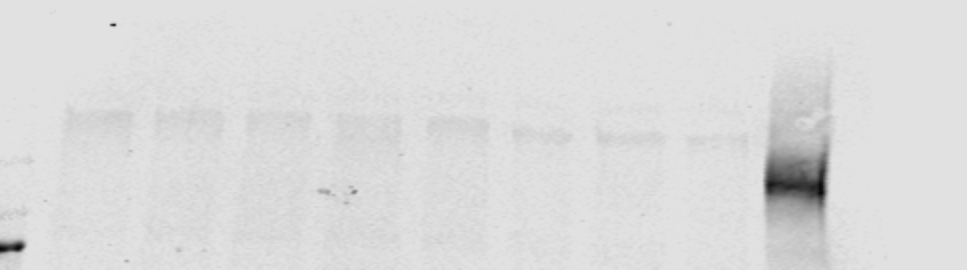

Supplement: Supplementary file 1 [file cancers-13-05644-s001.zip › Original Images of WB in Figure S1/Supplementary Figure 1C MDR1 Fusion Negative.tif]

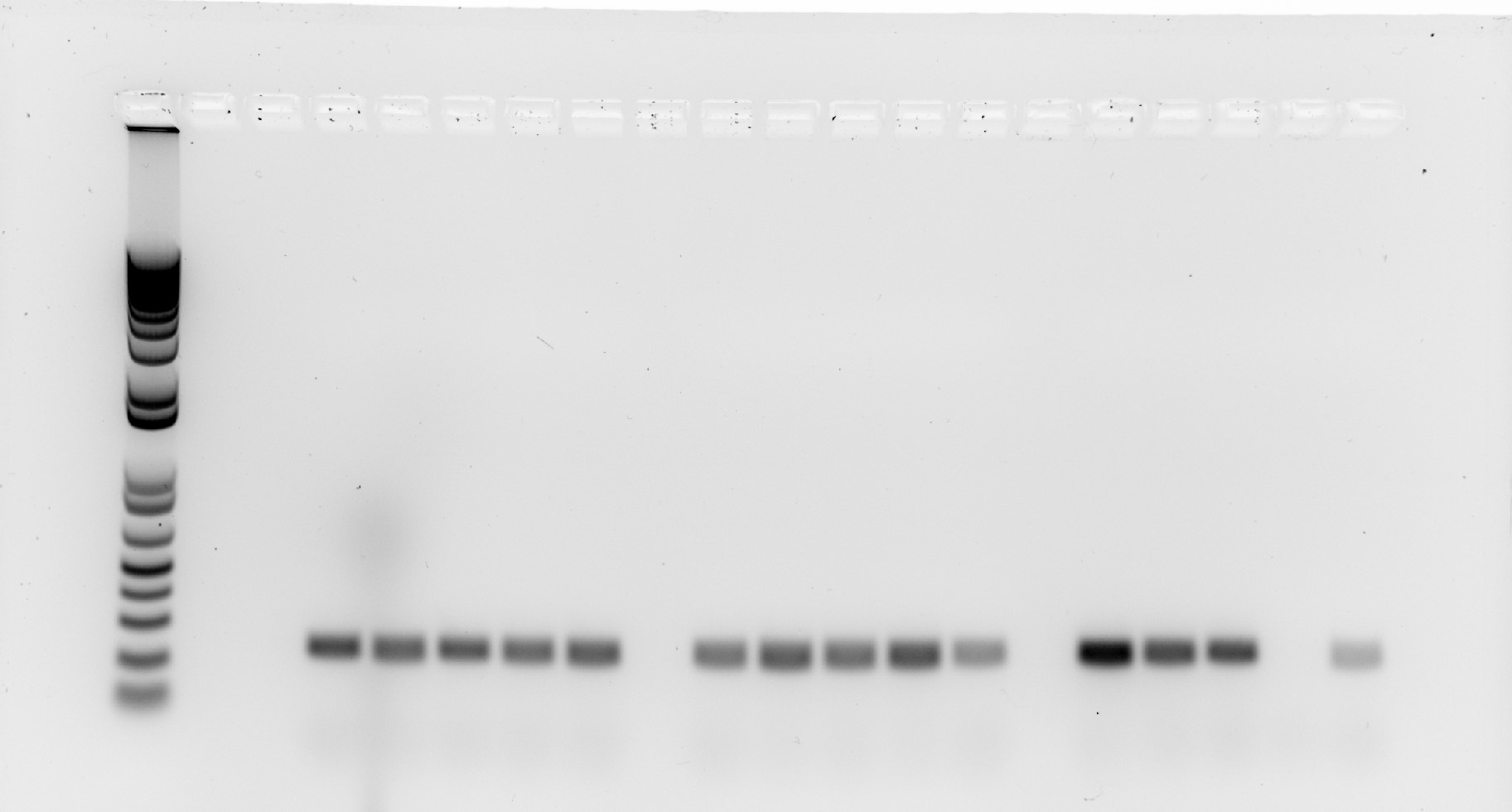

Supplement: Supplementary file 1 [file cancers-13-05644-s001.zip › Original Images of WB in Figure S1/Supplementary Figure S1A HPRT.jpg]

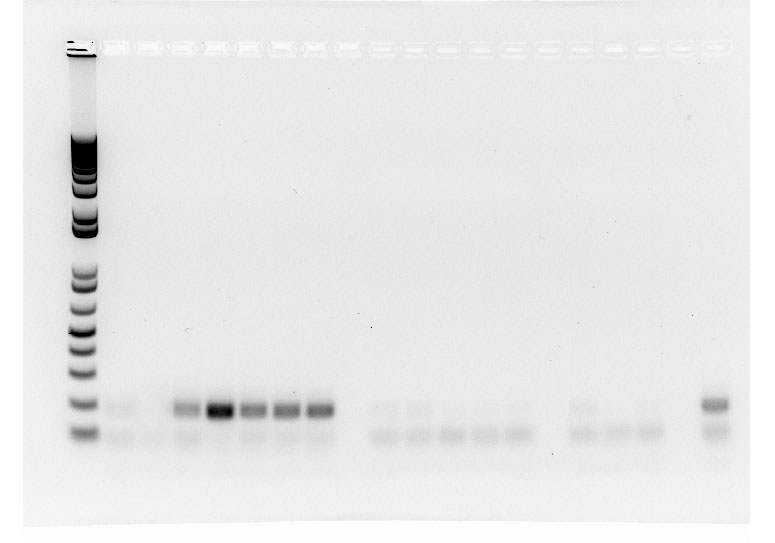

Supplement: Supplementary file 1 [file cancers-13-05644-s001.zip › Original Images of WB in Figure S1/Supplementary Figure S1A_SLC25A40.jpg]
