# Supplementary material for: Phenotypic Consequences of SLC25A40-ABCB1 Fusions beyond Drug Resistance in High-Grade Serous Ovarian Cancer
Source: Cancers (Basel). 2021 Nov 11;13(22):5644. doi: 10.3390/cancers13225644 (PMC8616176; doi:10.3390/cancers13225644)
Supplement: Supplementary file 1 [file Supplementary Figure S9 FDA Cell Cycle Profiles.pdf › Supplementary Figure S9 FDA Cell Cycle Profiles.pdf]

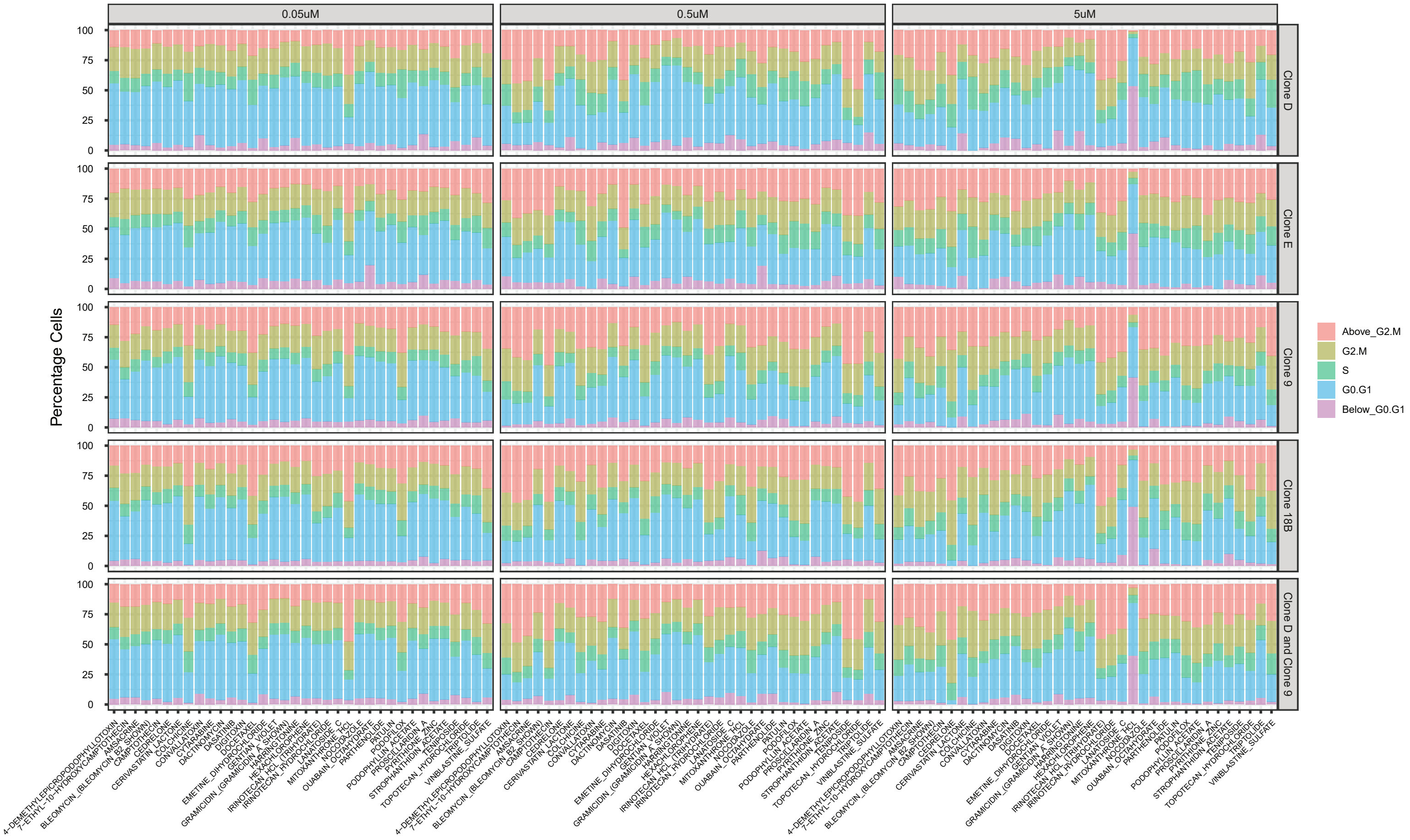

Supplementary Fig. S9: FDA agents primarily induce G0-G1 and above G2-M arrest in AOCS18.5 clones

Cell cycle profiles of AOCS18.5 fusion-negative (Clone D, Clone E), fusion-positive (Clone 9, Clone 18B) and 50:50 mixed (Clone D: Clone 9) cellular populations following 72hr treatment with 36 different FDA approved agents (0.05, 0.5 and 5µM). Percentage cells in specific cell cycle phase shown. Data determined through DAPI staining from two replicate wells.
